# Computationally efficient meta-analysis of gene-based tests using summary statistics in large-scale genetic studies

**DOI:** 10.1038/s41588-025-02390-0

**Published:** 2025-11-12

**Authors:** Tyler A. Joseph, Joelle Mbatchou, Arkopravo Ghosh, Anthony Marcketta, Christopher E. Gillies, Jing Tang, Priyanka Nakka, Xinyuan Zhang, Jack A. Kosmicki, Carlo Sidore, Lauren Gurski, Regeneron Genetics Center, Tyler A. Joseph, Joelle Mbatchou, Arkopravo Ghosh, Anthony Marcketta, Christopher E. Gillies, Jing Tang, Priyanka Nakka, Xinyuan Zhang, Jack A. Kosmicki, Carlo Sidore, Lauren Gurski, Maya Ghoussaini, Manuel A. R. Ferreira, Gonçalo Abecasis, Jonathan Marchini, Maya Ghoussaini, Manuel A. R. Ferreira, Gonçalo Abecasis, Jonathan Marchini

**Affiliations:** Regeneron Genetics Center, Tarrytown, NY USA

**Keywords:** Genome-wide association studies, Software

## Abstract

Meta-analysis of gene-based tests using single-variant summary statistics is a powerful strategy for genetic association studies. However, current approaches require sharing the covariance matrix between variants for each study and trait of interest. For large-scale studies with many phenotypes, these matrices can be cumbersome to calculate, store and share. Here, to address this challenge, we present REMETA—an efficient tool for meta-analysis of gene-based tests. REMETA uses a single sparse covariance reference file per study that is rescaled for each phenotype using single-variant summary statistics. We develop new methods for binary traits with case–control imbalance, and to estimate allele frequencies, genotype counts and effect sizes of burden tests. We demonstrate the performance and advantages of our approach through meta-analysis of five traits in 469,376 samples in UK Biobank. The open-source REMETA software will facilitate meta-analysis across large-scale exome sequencing studies from diverse studies that cannot easily be combined.

## Abstract (web summary)

REMETA is a method for meta-analyzing gene-based associations using summary statistics, integrating with REGENIE and improving computational efficiency for large studies with many phenotypes.

## Main

Over the past 10 years large-scale exome-wide association studies (ExWAS) have proven effective at finding genes associated with disease^1^. By focusing on protein-altering variants, ExWAS often provide interpretable association signals that can help identify therapeutic targets and guide the treatment of disease. For example, the discovery of rare protein-coding variants in *GPR75* associated with lower body mass index (BMI) suggest inhibiting *GPR75* as a potential therapeutic strategy for obesity^2^. Similarly, rare protein-coding variants in *CIDEB* are associated with protection against liver disease suggesting *CIDEB* as a therapeutic target^3^.

When combined with array genotyping followed by imputation, ExWAS has association power comparable to that of whole-genome sequencing for single-variant and gene-based tests^4^.

As most protein-altering variants are rare, ExWAS try to improve power by combining variants in a gene into gene-based tests. Different tests will be better powered to detect an association depending on the genetic architecture of a trait. ‘Burden tests’ are used widely for this purpose and have good power when causal variants alter gene function in the same effect direction^5,6^. Variance component tests model the distribution of effect sizes and can have more power when causal variants act in different directions^7,8^. Alternatively, methods that combine single-variant tests into a single *P* value can be particularly powerful when there are only a small number of causal variants^9^. Since the true genetic architecture is unknown, several types of gene-based tests can be combined into a single omnibus test to correct for multiple testing^10,11^. A key component of this approach is the use of variant annotations to group protein-damaging variants in each gene to test. Predicting the effect of a variant on protein structure is an active area of research, and a variety of annotation resources exist^12–14^.

Meta-analysis of ExWAS across datasets of diverse ancestry also increases power for new drug target discovery. Effect size meta-analysis, also known as inverse-variance weighted meta-analysis, can be used to combine burden tests across studies using estimates of effect sizes and their standard errors. For other tests that do not produce effect size estimates, such as variance component methods, a variety of *P* value meta-analysis methods are available for combining across studies. These approaches, which we refer to as standard meta-analysis, are simple and quick to apply, and produce reasonable results^2,3^.

However, meta-analysis of gene-based tests can be challenging if the contributing studies use different annotation resources or different criteria to group variants for testing. Inconsistencies in the variants included in tests can complicate downstream interpretation and analysis. For example, many tests use an allele frequency threshold to include a variant in a gene set. Differences in allele frequencies across studies can change the variants selected in each study. In addition, annotation resources evolve as new approaches are developed. Updating a meta-analysis to use new annotations requires re-analysis of all genes across all studies and traits, which can be costly and time consuming.

These problems can be alleviated by using gene-based tests that leverage single-variant summary statistics. Some gene-based tests can be calculated from appropriately scaled effect size estimates (that is, score statistics) of individual single variants in a gene, and measures of linkage disequilibrium (LD), or covariance, between those summary statistics^15,16^. Summary statistics can be combined across studies for meta-analysis, allowing for fine-scale control of the variants included in a test without any repeated association analysis (that is, no need to go back to the raw genetic or phenotypic data). Gene-based testing from summary statistics can be used to test each gene marginally, or conditional upon a set of specified variants. The software programs RAREMETAL^16^ and metaSTAAR software^17^ implement this approach. However the LD information required varies according to the exact set of participants and trait being analyzed. Thus, an LD-like matrix must be computed for each study and trait—which can be challenging to calculate, store and manipulate for studies with large numbers of traits.

In this paper, we describe a new approach to this problem that has several key properties. First, we show that sparse reference LD files derived from all participants in a study can be substituted accurately for the exact LD files for a given study, even if it includes just a subset of participants. Such reference LD matrices can be precalculated once for a study and used for subsequent analyses, which can substantially reduce the compute and storage requirements of gene-based tests. This also greatly facilitates sharing of LD files between groups of researchers since only one LD file needs be shared per study, and not per phenotype and study.

Second, we have developed a compact per-chromosome binary file format for efficiently storing and sharing per study LD matrices that are required for gene-based tests. This format handles both marginal and conditional testing scenarios and is indexed to allow fast access to the LD information of any gene.

Third, since *P* values are not sufficient for follow up interpretation of gene-based tests, we develop an approximate method for calculating allele frequencies, genotype counts and effect size of burden tests from summary statistics.

Fourth, we extend the approach to handle binary trait meta-analysis of gene-based tests with high case–control imbalance and show that this is well calibrated.

Finally, for ease of use we have developed this approach in an open-source software package called REMETA^18^, which is designed to integrate seamlessly with the REGENIE software.

## Results

### Methods overview

**Fig. 1:**
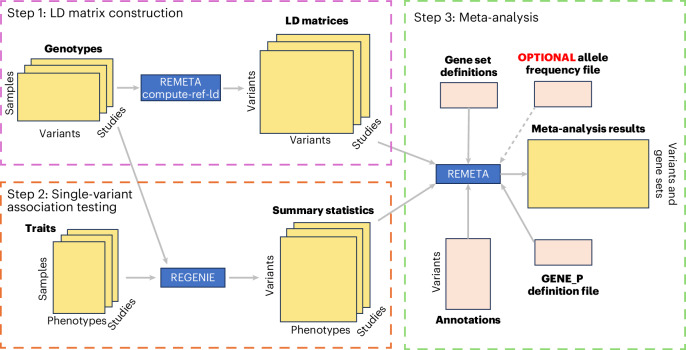
Overview of REMETA workflow. The REMETA workflow has three steps: (1) LD matrix construction in REMETA, (2) single-variant association testing with REGENIE and (3) meta-analysis. REMETA uses gene sets, variant annotations and an optional allele frequency file to construct gene-based tests from single-variant summary statistics and LD matrices during meta-analysis. *P* values across masks and tests are combined using ACAT into a single ‘GENE_P’ *P* value per gene to limit multiple testing. The GENE_P definition file defines which masks to include in GENE_P.

The REGENIE/REMETA workflow is applicable to the setting where there are *T* traits measured in *K* studies with array genotypes and whole-exome sequencing (WES) data at *P* genes, and proceeds by the following three steps (Fig. 1).

#### LD matrix construction

The first step constructs reference LD matrices for each study in REMETA. This step needs to be carried out only once for each study. Separate matrices for each of the *T* traits are not required, as with existing approaches^17^, which substantially reduces compute cost and storage. We developed a custom file binary file format for LD matrices that is indexed so that LD matrices for individual genes can be extracted quickly. The LD files can be constructed from just the WES dataset, which is sufficient for testing each gene marginally, or from a WES dataset and a file of imputed variants across the genome to compute gene-based tests conditional on GWAS loci.

#### Single-variant association testing

The second step involves running REGENIE Step 1 on the array genotypes for each study for the *T* traits with any appropriate covariates. This step accounts for relatedness, population structure and polygenicity. The polygenic scores produced are then used as additional covariates in REGENIE Step 2 where association testing of individual variants in the WES dataset is carried out for each phenotype. It is important to note that all polymorphic variants must be analyzed in this step, without any filter on minor allele count. Exclusion of any variants at this step will mean they cannot be included in any downstream gene-based test using REMETA. One key advantage of using REGENIE is that several traits can be run in parallel, which controls computational expense and can be simpler to run. We have added the flag–htp flag to Step 2 of REGENIE to produce more detailed summary statistic output required by REMETA.

#### Meta-analysis

The third step carries out gene-based meta-analysis using REMETA. The inputs to this step are the REGENIE summary statistic files for each trait and study, the REMETA LD files for each study, gene set and variant annotation files, and an optional list of variants to condition on. REMETA uses the reference LD files for each study, applies a scaling and transformation that based on the trait being meta-analyzed, and computes burden tests, optimal sequence kernel association test (SKATO) variance component tests and the aggregated Cauchy association test (ACATV), using a set of allele frequency bins specified by the user. Tests can also be combined into an overall ‘GENE P’ *P* value for each gene. Overall, this step provides users with fine-scale control of how meta-analysis is carried out.

### Approximate gene-based testing using per study LD

The gene-based tests we consider are constructed from the score statistics of single variants. For burden testing and SKATO, computing a *P* value requires the covariance matrix of the score statistics to account for the LD of the variants in the test (Supplementary Note 1). This matrix varies per trait, which can be cumbersome to compute and store across many traits. We therefore wanted to reduce the storage requirements from one matrix per trait to one matrix per study.

In the special case of an association model that tests one single-nucleotide polymorphism (SNP) with just an intercept as a covariate, the covariance matrix of the score statistics is equivalent to a rescaling of the covariance matrix of the genotypes in a test^15,19^. Therefore, a natural strategy is to compute the covariance of the genotypes once per study, then adjust it for the trait being analyzed. To make the adjustment, we store the variance of the score statistics computed per trait (Methods). Intuitively, the adjustment corrects for differences in sample size and phenotypic variance across traits. To further reduce the storage requirements the covariance matrix can be stored sparsely, keeping only entries between pairs of exome variants where ${r}^{2} > {10}^{-4}$ (adjustable through a command line parameter). We refer to the sparse covariance of the genotypes computed once per study as the ‘reference LD’ matrix, and the covariance of the score statistics as the ‘exact LD’ matrix. The goal is to assess how well *P* values computed using the reference LD matrix approximate those from the exact LD matrix.

**Extended Data Fig. 1:**
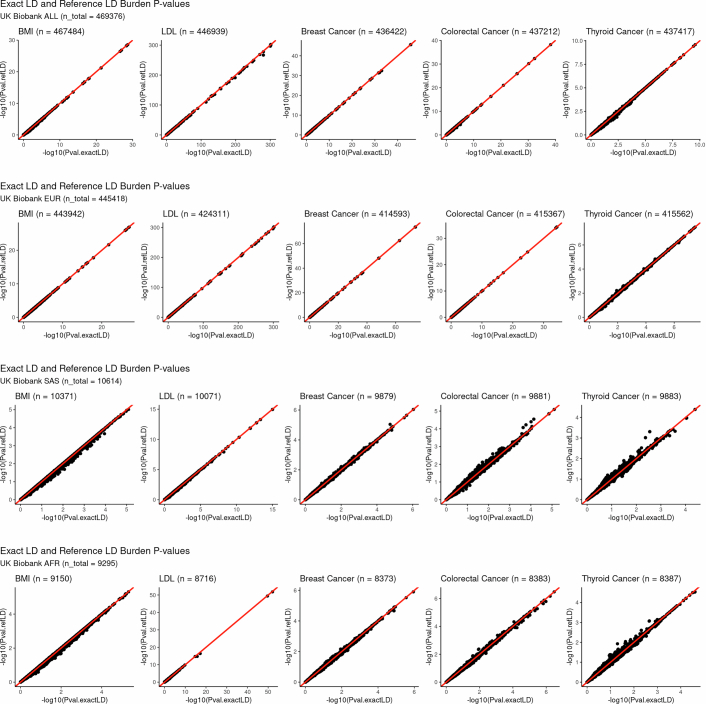
Scatterplots comparing p-values of burden tests computed using the exact covariance of the score statistics and the approximation using a reference LD panel. Each row corresponds to an ancestry, and each column corresponds to trait. In each case we computed reference LD matrices within each ancestry and compared *p*-values computed with reference LD (y-axis; Pval.refLD; sample sizes given by n_total) to those computed using the exact LD matrix (x-axis; Pval.exactLD). Two-sided p-values for burden tests are computed across 7 annotations categories at 5 allele frequency bins, resulting in 35 points in each scatterplot for each gene.

**Extended Data Fig. 2:**
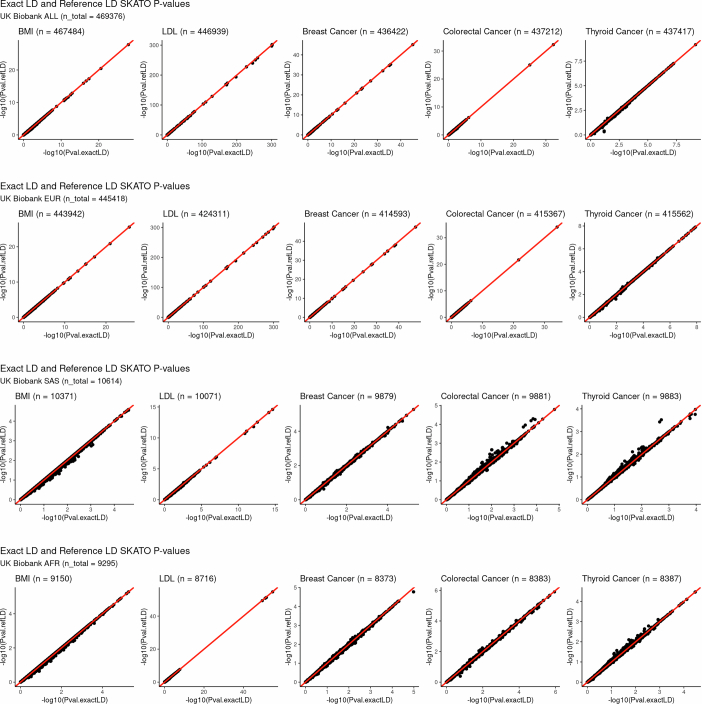
Scatterplots comparing SKATO p-values computed using the exact covariance of the score statistics and the approximation using a reference LD panel. Each row corresponds to an ancestry, and each column corresponds to trait. In each case we computed reference LD matrices within each ancestry and compared *p*-values computed with reference LD (y-axis; Pval.refLD; sample sizes given by n_total) to those computed using the exact LD matrix (x-axis; Pval.exactLD). SKATO is computed across 7 annotation categories using a 1% AAF cutoff, resulting in 7 points in each scatterplot for each gene.

We evaluated the performance of this approximation across a range of scenarios using five traits in the UK Biobank (UKB) (*n* = 469,376): BMI, low-density lipoprotein (LDL), breast cancer (case:control ratio, 1:25), colorectal cancer (case:control ratio, 1:69) and thyroid cancer (case:control ratio, 1:630). We found that approximate *P* values are accurate across a wide range of settings. Extended Data Fig. 1 (top row) shows the performance of the approximation when the reference LD is calculated in the full UKB samples (all *N* = 469,376) and used to calculate burden test *P* values (specifically the sum test^5^; Methods) for BMI (*N* = 467,484), LDL (*N* = 446,939), breast cancer (*N* = 436,422), colorectal cancer (*N* = 437,212) and thyroid cancer (*N* = 437,417). As LD can vary among genetic ancestries, we repeated this experiment in different subsets of UKB (Extended Data Fig. 1; remaining rows). All cases showed strong agreement among *P* values. Extended Data Fig. 2 shows analogous results for the SKATO test.

The previous scenarios had the property that the sample sizes of the trait being analyzed were close to the size of the reference panel. We hypothesized that the approximation might break down when the subset was small and/or the ancestry composition of the subset was different that of the full reference panel. Supplementary Fig. 1 shows the performance of the approximation when reference LD is calculated in the full UKB samples (all *N* = 469,376) and used to calculate sum test *P* values in: a random subset about one-third of the size, in samples from Europe (EUR), South Asia (SAS) and Africa (AFR). In all cases the agreement is very good. Supplementary Fig. 2 shows analogous results for the SKATO test.

One possible explanation for the strong concordance between *P* values is that there is little or no LD between the exome variants in a test. In the absence of LD between variants, the reference LD matrix is equal to the exact LD matrix, and there would be no need to store LD matrices per study. Therefore, we compared *P* values computed using the exact LD matrix to *P* values computed by ignoring any LD between variants (Supplementary Fig. 3). When LD between variants is ignored, test statistics at many genes are unaffected, but we observed inflation in the *P* values of some tests at some genes, suggesting that LD between variants is required to have well-calibrated tests. When carrying out conditional tests of association, using LD is crucial.

### Estimating genotype counts, allele frequencies and effect sizes

**Extended Data Fig. 3:**
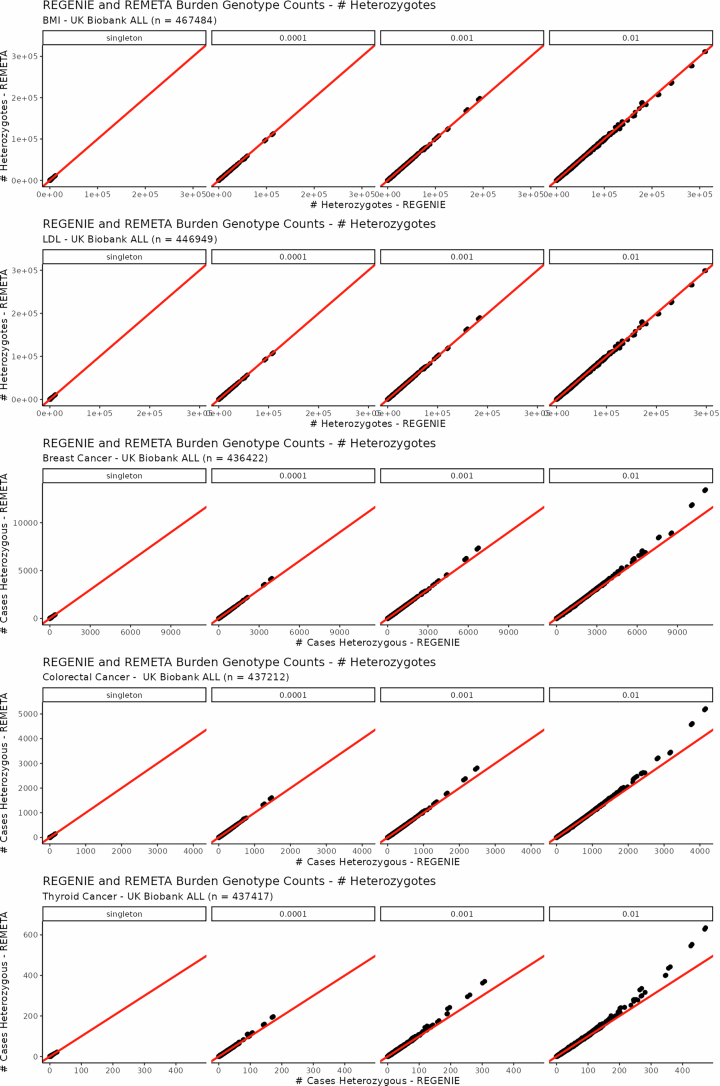
Scatterplot comparing heterozygote genotype counts of burden masks computed by REGENIE and estimated by REMETA. Each row corresponds to a trait, and each column corresponds to an AAF bin for a burden test. Each panel includes burden masks computed across 7 annotation categories. For quantitative traits, heterozygote genotypes counts in the whole sample are displayed. For binary traits, heterozygote genotype counts in cases are displayed.

**Extended Data Fig. 4:**
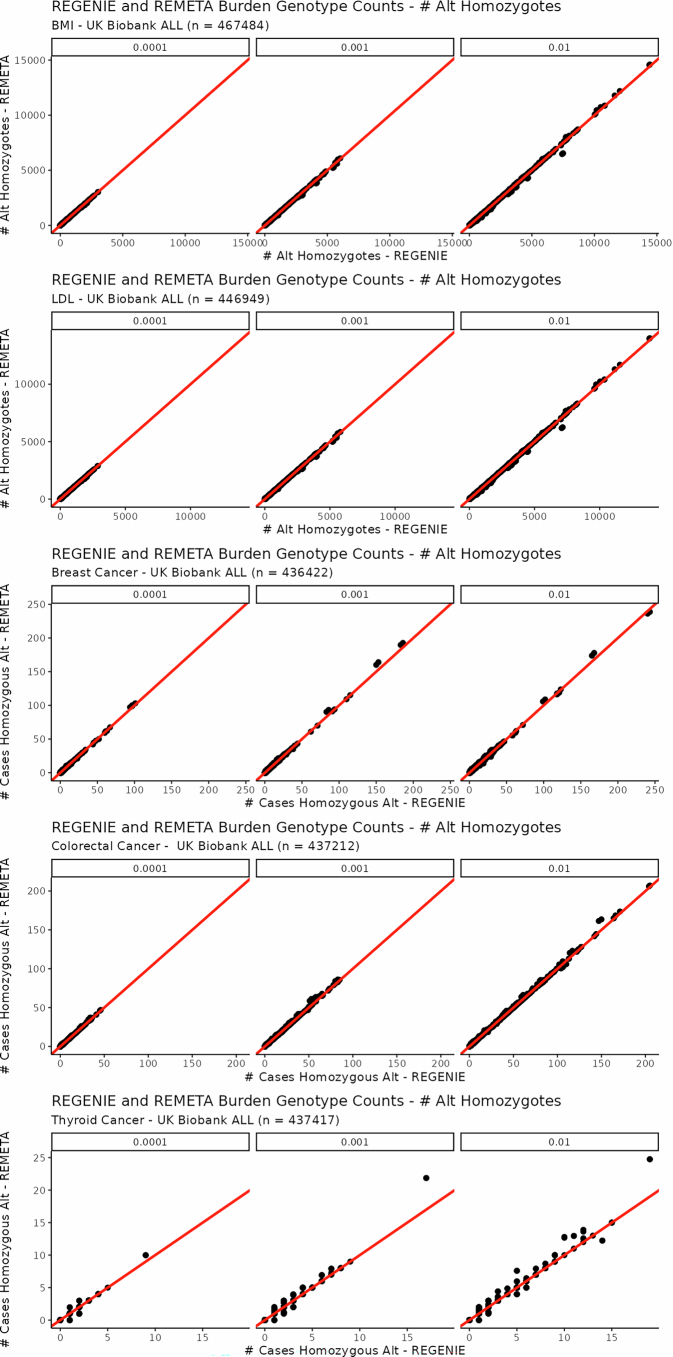
Scatterplot comparing homozygous alternate genotype counts of burden masks computed by REGENIE and estimated REMETA. Each row corresponds to a trait, and each column corresponds to an AAF bin for a burden test. Each panel includes burden masks computed across 7 annotation categories. For quantitative traits, homozygous alternate genotypes counts in the whole sample are displayed. For binary traits, homozygous alternate genotype counts among cases are displayed.

**Extended Data Fig. 5:**
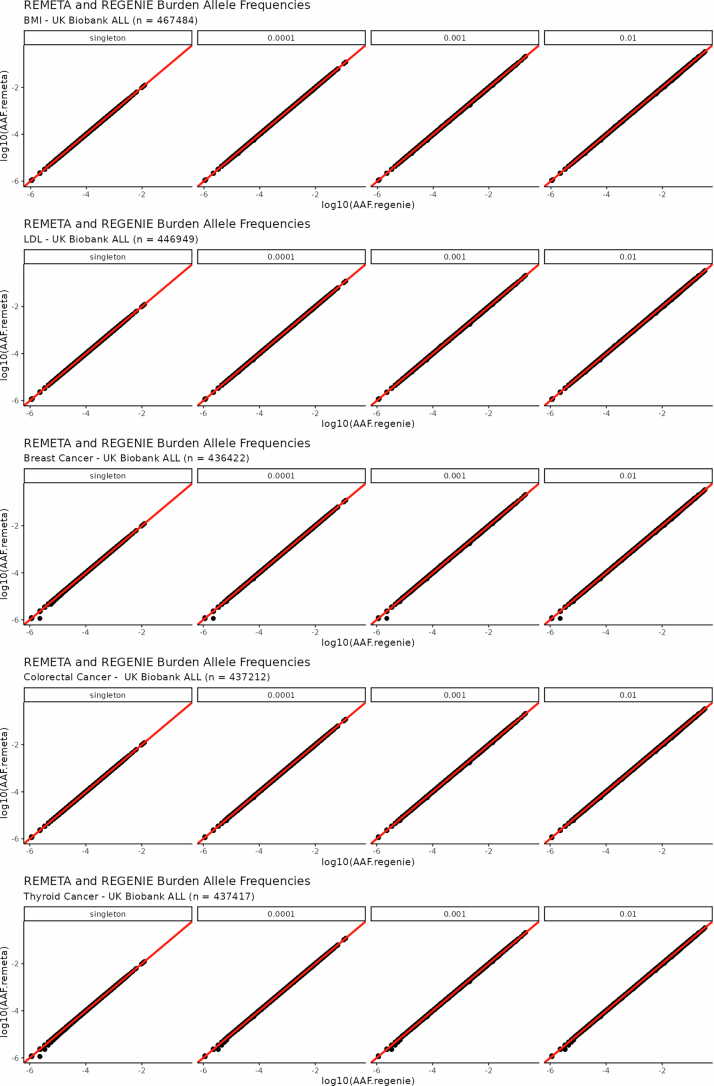
Scatterplot comparing allele frequencies of burden masks computed by REGENIE and estimated by REMETA. Each row corresponds to a trait, and each column corresponds to an AAF bin for a burden test. Each panel includes burden masks computed across 7 annotation categories. The x-axis in each figure is the exact AAF computed in REGENIE, while the y-axis is AAF estimated by REMETA.

**Extended Data Fig. 6:**
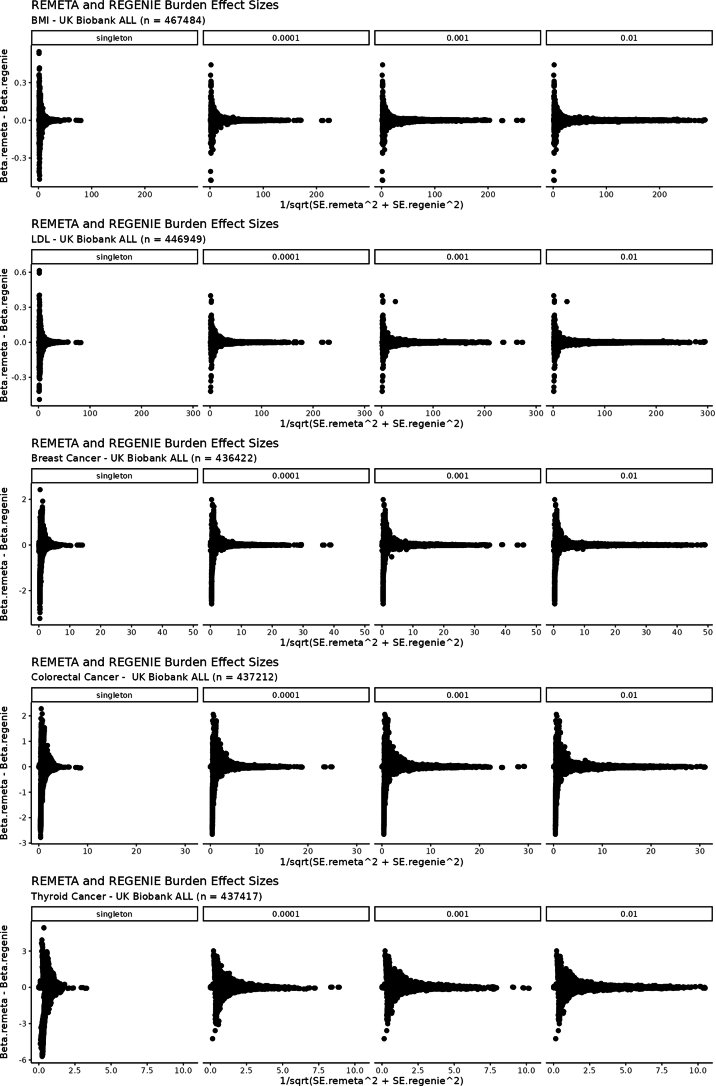
Comparison of effect size estimates from the sum test and the collapsing variant test. Differences in effect size estimates between the sum test (Beta.remeta) and collapsing variant test (Beta.regenie). Note that the standard error of the difference Beta.remeta – Beta.regenie is $\sqrt{{\rm{S}}{{\rm{E}}}^{2}.{\rm{remeta}}+{\rm{S}}{{\rm{E}}}^{2}.{\rm{regenie}}}$. Each panel includes burden masks computed across 7 annotation categories.

Genotype counts, allele frequencies and effect sizes are crucial information when interpreting burden test associations. The default burden test in REGENIE is performed using the collapsing variant test, while REMETA uses the sum test^5,20^ to construct burden masks because it can be computed from single-variant summary statistics. Despite differences in how the tests are computed, the *P* values from both tests are concordant (Supplementary Figs. 4 and 5), motivating us to use the sum test as an approximation to the collapsing variant test. We derived new methods to estimate genotype counts and allele frequencies of the burden masks for collapsing variant test from the single-variant frequencies and reference LD (Methods) and show that our estimator accurately approximates allele frequencies and genotype counts computed in REGENIE (Extended Data Figs. 3–5). Heterozygote genotype counts were least accurate for missense masks in MUC16 and TTN, which had an alternate allele frequency (AAF) > 20% and more than 10,000 variants in a mask, while genotype counts were particularly accurate for burden masks comprising pLoF variants (Supplementary Figs. 6 and 7). A simpler approach that sums genotype counts of mask variants overestimates burden mask genotype counts (Supplementary Fig. 8). Finally, we derived estimates of the effect size for the sum test (Methods), and show that it accurately approximates the collapsing variant test estimate (Extended Data Fig. 6).

### Computational and storage costs

**Fig. 2:**
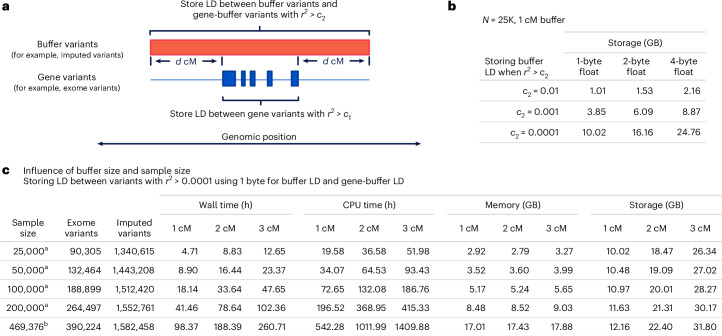
Resource requirements to generate LD matrices for conditional analysis. Experiments were performed using chr. 20 in UKB. **a**, Depiction of REMETA’s strategy for computing LD matrices. **b**, How the *r*^2^ threshold influences LD file sizes for three floating-point storage types. **c**, Wall time, CPU time, memory usage and storage requirements to generate LD matrices in subsets of UKB. ^a^Experiments run using 16 cores. ^b^Experiments run using 32 cores.

Reassured that gene-based tests can be approximated from sparse per study LD matrices, we developed a compact file format to store LD matrices. LD matrices can be computed for either marginal testing by only including exome variants, or for conditional analysis by including exome variants and imputed variants in a buffer region around each gene (Fig. 2a). Because generating LD matrices between large numbers of variants can be compute and storage intensive, we explored several options to improve scalability. To speed up compute time, REMETA performs matrix multiplication using single-precision floating-point numbers instead of double-precision floating-point numbers. Using Advanced Vector Extensions (AVX) for x86 architectures, eight single-precision floating-point numbers can be multiplied simultaneously, doubling the speed of matrix multiplication compared to four double-precision floating-point numbers. To reduce LD file sizes, REMETA supports storing three floating-point types: four-byte single-precision, two-byte and one-byte floating-point numbers (Fig. 2b). The output LD matrices are compressed using HTSlib with the DEFLATE library and indexed per gene to quickly query LD matrices per gene.

Figure 2c illustrates the scalability of LD matrix generation in REMETA with varying sample and buffer sizes in subsets of the UKB. REMETA’s main constraint is compute time, which approximately doubles for each doubling in sample size and increases linearly with buffer size. In contrast, memory and storage requirements increase more slowly with sample size. Across all experiments REMETA used <18 Gb of memory. LD file sizes were comparable across sample sizes, reflecting the similar numbers of imputed variants in each subset.

We benchmarked LD matrix generation for marginal analysis against MetaSTAAR by computing per gene LD matrices in subsets of UKB (Table 1). Because MetaSTAAR computes LD matrices per trait, at each sample size we simulated a null quantitative trait from a standard normal distribution without covariates. We compared central processing unit (CPU) time, memory usage and file storage between REMETA and MetaSTAAR. At 200,000 samples, we found that REMETA was >2.5× faster than MetaSTAAR, used 77% less memory and generated files that were 56% smaller than those generated by MetaSTAAR. Overall, LD matrices for marginal analysis are small and quick to compute because they require only the LD between exome variants in a gene. For conditional analysis, the comparison between MetaSTAAR and REMETA is not so straightforward. MetaSTAAR requires a user to specify a set of variants to condition on before the calculation of the LD matrices.

**Table 1:** Resource requirements to generate LD matrices for marginal analysis

Sample size	CPU time (min)		Memory (Gb)		Storage (Mb)	
	REMETA	MetaSTAAR	REMETA	MetaSTAAR	REMETA	MetaSTAAR
25,000	0.95	23.55	1.34	2.77	1.16	2.67
50,000	2.93	29.81	2.26	5.41	1.41	3.10
100,000	9.15	41.15	3.95	12.33	1.73	3.86
200,000	28.06	75.16	7.17	30.63	2.16	4.93

Per gene LD matrices were computed on chr. 20 using exome variants in subsets of UKB. Note that MetaSTAAR requires an LD matrix per trait in each study while REMETA requires only a single LD matrix per study. Experiments were run using a single core.

### Conditional analysis

Conditioning gene-based tests on nearby common variants can help identify which associations are shadows of common-variant signals due to LD. In conditional analysis from summary statistics, the variants available to condition on depends on the size of the buffer region around each gene and the *r*^2^ threshold to store elements in the LD matrix. Both parameters influence the size of the matrices stored. We therefore asked: how large do LD matrices need to be to effectively condition out most common-variant signals?

**Fig. 3:**
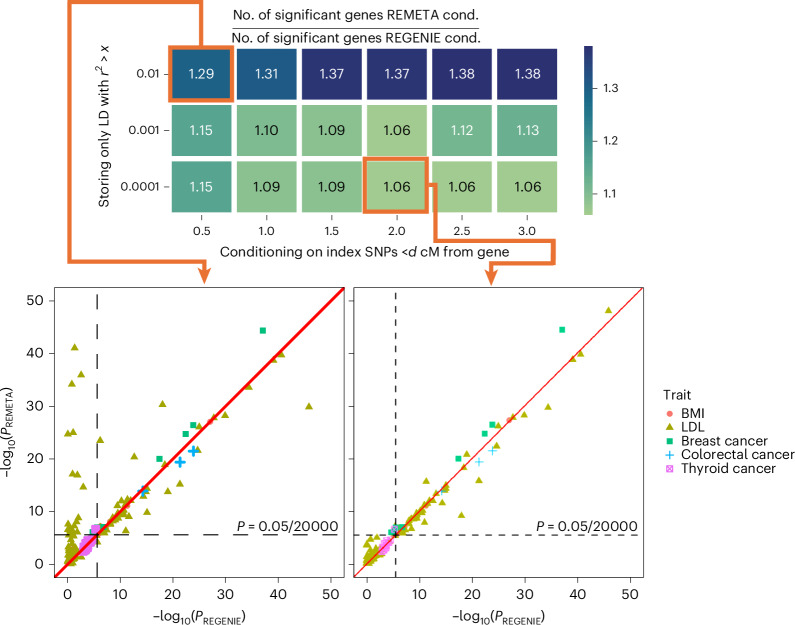
Evaluation of LD buffer size and *r*^2^ threshold required to condition out common-variant signals. Number of significant gene–trait pairs from REMETA conditional analysis (REMETA cond.) compared to REGENIE conditional analysis (REGENIE cond.). Two-sided *P* values from burden tests, SKATO and ACATV are combined into a single GENE_P *P* value per gene that corrects for multiple testing. REMETA conditional analysis is performed from summary statistics while varying the buffer size and *r*^2^ threshold. REGENIE conditional analysis is performed by including all index SNPs on a chromosome as covariates. At *d* = 2 and *r*^2^ > 0.0001 REMETA conditions out most common-variant signals. The remaining significant gene–trait pairs are near the significance cutoff.

We re-analyzed all 157 ExWAS significant (*P* < $\frac{0.05}{20,000}$) GENE_P^21^ (Methods) associations found among the five traits in UKB. We computed gene-based tests conditional on index SNPs computed by LD clumping common variants for each trait (minor allele frequency > 0.01; *r*^2^ > 0.1), and compared *P* values from REMETA to *P* values from REGENIE. For all traits except LDL REMETA’s conditional *P* values were similar across buffer sizes and *r*^2^ thresholds (Fig. 3 and Supplementary Fig. 9). Conditional analysis for LDL, which had the most index SNPs and some very strong common-variant associations, was sensitive to both (Fig. 3a). We observed similar results when conditioning on SNPs with the top conditional posterior inclusion probability (cPIP) from each credible set using variants fine-mapped with SuSiE^22,23^ (Supplementary Fig. 10).

Because LD matrices for conditional analysis can be large, we asked whether REMETA could perform conditional analysis using LD matrices for marginal analysis combined with single-variant summary statistics conditional on nearby common variants. We computed summary statistics in REGENIE conditional on top SNPs from variants fine-mapped with SuSIE, and provided those summary statistics to REMETA. We then compared GENE_P *P* values from REMETA’s gene-based test to REGENIE’s gene-based tests conditioned on the same set of variants. The *P* values from both methods were highly concordant (Supplementary Fig. 11), indicating that this could be used as an alternate approach for conditional analysis.

### Type 1 error and unbalanced binary traits

For unbalanced binary traits using a normal approximation for the score statistic can lead to inflated type 1 error. In these instances, a saddlepoint approximation (SPA) has proven an effective strategy to help calibrate *P* values. We evaluated two types of SPA from summary statistics to control type 1 error: an SPA applied per mask and an SPA applied per variant (Methods). For the mask-based SPA, we used the observations that the sum test in REMETA approximates the collapsing variant test REGENIE, and that we can estimate genotype counts of the collapsing variant test from the variants in the test and their LD. This allows us to apply an SPA^24^ for the collapsing variant test and use it to compute a calibration factor to rescale the LD matrix. For the variant-based SPA, we used a similar strategy to Park et al.^25^. We apply a per-variant SPA and use it to compute a calibration factor to adjust variance of the score statistic for each variant.

We evaluated how well each SPA controlled type 1 error by simulating null traits in three equally sized subsets of UKB across a range of case:control ratios (Extended Data Table 1). We found that applying a mask-based SPA helped control type 1 error in burden tests, whereas applying both SPAs was sometimes too conservative. In contrast, both SPAs were required to control type 1 error for SKATO. For ACATV, we found that applying the variant-based SPA helped control type 1 error. Our simulations show that REMETA provides good type 1 error control across traits.

### Application to meta-analysis of UKB

We applied REMETA to carry out gene-based meta-analysis of three equally sized subsets of UKB with ~156,000 samples each (*N* = 469,376 total) for BMI, LDL, breast cancer, colon cancer and thyroid cancer. For each subset, we generated LD matrices with REMETA and single-variant summary statistics with REGENIE.

**Fig. 4:**
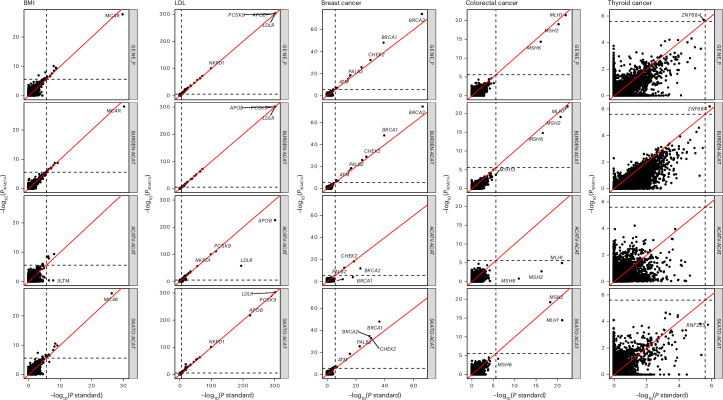
Comparison of −log_10_*P* values between REMETA to standard meta-analysis across five traits. Each point is a single gene, where two-sided *P* values per gene have been combined using ACAT that corrects for multiple testing. Four types of gene-based tests are compared: GENE_P, burden, ACATV and SKATO. *GENE_P: Omnibus test combining *P* values from BURDEN-ACAT, ACATV-ACAT and SKATO-ACAT using ACAT. *BURDEN-ACAT: ACAT of burden meta-analysis *P* values. *ACATV-ACAT: ACAT of ACATV meta-analysis *P* values per gene. *SKATO-ACAT: ACAT of SKATO meta-analysis *P* values per gene. Dashed lines correspond to a *P* value of 2.5 × 10^−6^.

**Fig. 5:**
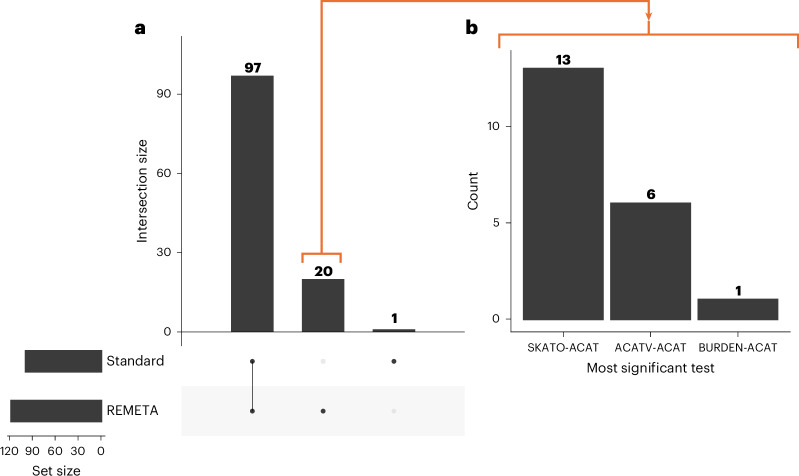
Significant gene–trait associations in REMETA and standard meta-analysis. **a**, Number of GENE_P significant gene–trait associations $(P < \frac{0.05}{20,000\times 5})$ from REMETA and standard meta-analysis across five traits in UKB. **b**, Most significant test included in GENE_P for each of 22 gene–trait pairs that were significant in REMETA alone.

We compared the performance of REMETA to the standard meta-analysis approach that combines effect sizes for burden tests (effect size meta-analysis) and combines *P* values (*P* value meta-analysis (PVMA)) for SKATO and ACATV tests. The overall omnibus GENE_P *P* values^21^ for both models are largely consistent (Fig. 4 top row), with REMETA identifying 117 significant gene–trait associations ($P < \frac{0.05}{20,000\times 5}$) compared to 98 significant gene–trait associations identified by standard meta-analysis (Fig. 5a and Supplementary Table 1). Notably, only one significant gene–trait pair in standard meta-analysis was not significant in REMETA (*IGSF23* with LDL; standard meta-analysis GENE_P = 4.4 × 10^−7^, REMETA GENE_P = 6.2 × 10^−7^). To better understand which tests contributed to the 20 gene–trait pairs identified by REMETA alone, we counted the most significant test for each pair (Fig. 5b). SKATO-ACAT was the most significant test for 13 gene–trait pairs, followed by ACATV-ACAT for eight pairs and BURDEN-ACAT for one pair, suggesting that SKATO and ACATV meta-analysis from summary statistics is more powerful than SKATO and ACATV meta-analysis using PVMA.

For ACATV-ACAT, we observed associations stronger associations in standard meta-analysis than REMETA between *LDLR* in LDL, and *ATM*, *BRCA1* and *BRCA2* in breast cancer, and *MSH2*, *MSH6* and *MLH1* in colorectal cancer (Fig. 4 third row). These associations are driven by collapsing low minor allele count variants into a single meta-variant in REGENIE and including them in ACATV, whereas we did not collapse variants in REMETA. However, signals driven by variant collapsing are similar to burden signals, so these genes get picked by other tests.

**Extended Data Fig. 7:**
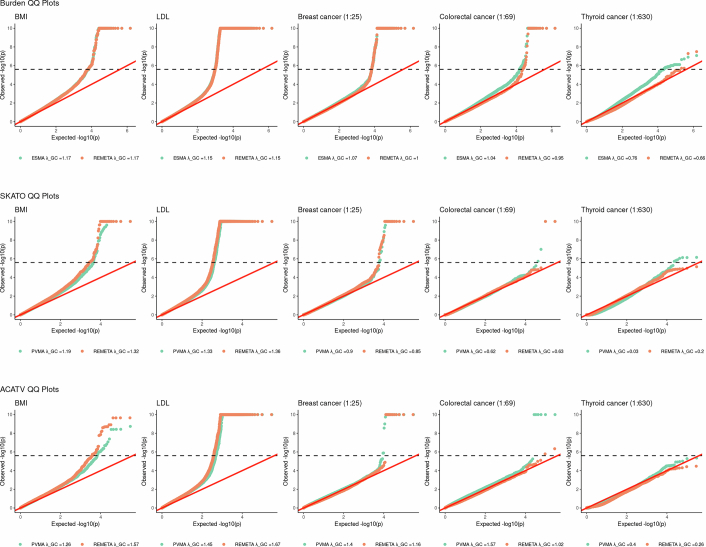
QQ plots from a meta-analysis of 3 equal sized subsets of UK Biobank. QQ plots for burden tests (top), SKATO (middle), and ACATV (bottom) across 5 traits in a meta-analysis of UK Biobank. λ_GC: genomic control.

For BURDEN-ACAT, we observed differences in *P* values between *BRCA1* and *BRCA2* in breast cancer, and *MLH1*, *MSH2* and *MSH6* in colorectal cancer (Fig. 4 second row). These are probably due to differences in type 1 error control between REMETA and standard meta-analysis (Extended Data Fig. 7). QQ plots across binary traits suggest that burden tests in REMETA have better type 1 error control than standard meta-analysis. Burden tests in REMETA were similarly calibrated to burden tests in REGENIE (Supplementary Figs. 12–14).

We repeated our meta-analysis of all five traits using three unequal subsets of UKB (a 1:2:3 ratio). The results were consistent with the meta-analysis of equal size subsets (Supplementary Fig. 15).

### Fast re-analysis using new variant annotations

To illustrate the fast and flexible properties of REMETA, we re-analyzed gene-based tests using ESM-1v protein language model annotations of missense variants^14^. We benchmarked how long re-analysis would take in REGENIE using the raw genotype and phenotype data, compared to REMETA using the summary statistics and LD for chr. 20 in BMI. Re-analysis took 162.9 CPU min (~54.3 CPU min per batch) in REGENIE and 7.94 CPU min in REMETA.

## Discussion

In this study, we proposed REMETA as a computationally efficient approach to meta-analysis of gene-based tests in ExWAS. The software integrates seamlessly with the REGENIE software used widely for carrying out individual study ExWAS. Two aspects of REMETA allow it to scale to several large studies such as UKB. First, REMETA uses a single LD matrix computed per study instead of one LD matrix per trait. We showed under a range of scenarios that one LD matrix per study is sufficient to perform gene-based testing from summary statistics. Second, REMETA efficiently computes and stores per study LD matrices for each gene. To aid in the interpretation of gene-based association results, we developed methods to estimate the allele frequencies, genotype counts and effect sizes of burden tests from single-variant summary statistics, whereas existing approaches have been able to provide only *P* values.

There are also limitations to the REMETA approach. For example, any update to the phenotypes, or changes to the covariates, requires recomputing single-variant summary statistics from the original data in REGENIE. It may be possible to incorporate new covariates through adjusting the reference LD matrix, although we leave this for future work. Furthermore, although we showed that LD between variants was not critical for marginal testing, accurate LD is important for conditional analysis and estimating genotype counts. Consequently, the size of the LD files for conditional analysis were large compared to those for marginal analysis.

The REMETA software tools and framework will facilitate meta-analysis across biobank datasets that cannot easily be brought together through sharing of appropriate summary statistic files. For example, the apparent move towards biobank datasets being hosted on distinct trusted research environments such the UKB RAP and the AllofUs Researcher Workbench means that these datasets are highly unlikely ever to be combined in a single environment. Centralized calculation of reference LD files for such studies that are then shared freely with researchers would greatly facilitate the ease with which meta-analysis across studies could occur.

## Methods

### Computing gene-based tests from summary statistics

We considered three gene-based tests that can be computed from single-variant summary statistics: the weighted sum test (WST), SKATO and ACATV. For a single gene-based test in a single study, the setup is as follows. For a sample of *n* individuals, let **y** be the *n* × 1 vector of phenotypes, *G* the *n* × *p* genotype matrix of variants in the test, and **w** a *p* *×* 1 vector of variant weights.

Both the WST and SKATO can be computed from the score statistics^20^ (using linear regression for quantitative traits and logistic regression for binary traits) of the variants included in the test and their covariance (Cov):

$$\bf{S}={G}^{T}({\bf{y}}-\hat{\boldsymbol \mu})$$

$$\mathrm{Cov}\left(\bf{S}\right)={G}^{T}\mathrm{Cov}(\,\bf{y}-\hat{\boldsymbol{\mu} }) G$$

where ${\hat{\boldsymbol{\mu}}}$ is estimated under the null hypothesis.

The test statistic for WST is

$${Q}_{{\rm{W}}{\rm{S}}{\rm{T}}}={\left(\mathop{\sum }\limits_{j=1}^{p}{w}_{j}{S}_{j}\right)}^{2}$$

where the ${w}_{j}$ are prespecified variant weights. We refer to the special case where ${w}_{j}=1$ as simply the ‘sum-test.’ Then

$$\frac{{Q}_{{\rm{W}}{\rm{S}}{\rm{T}}}}{\bf{w}^{T}{\rm{C}}{\rm{o}}{\rm{v}}(\bf{S})\bf{w}}\sim {\chi }^{2}(1)$$

The test statistic for SKATO is

$${Q}_{{\rm{S}}{\rm{K}}{\rm{A}}{\rm{T}}-{\rm{O}}}=(1-\rho ){Q}_{{\rm{S}}{\rm{K}}{\rm{A}}{\rm{T}}}+\rho {Q}_{{\rm{W}}{\rm{S}}{\rm{T}}}$$

where

$${Q}_{{\rm{S}}{\rm{K}}{\rm{A}}{\rm{T}}}=\mathop{\sum }\limits_{j=1}^{p}{w}_{j}^{2}{S}_{j}^{2}$$

The test statistic ${Q}_{{\rm{S}}{\rm{K}}{\rm{A}}{\rm{T}}-{\rm{O}}}$ follows a mixture of chi-square distribution. Similar to the original SKATO method, we compute *P* values for SKATO using a series of values for $\rho \in (\mathrm{0,0.01,0.04,0.09,0.16,0.25,0.5,1})$. However, instead of taking the minimum *P* value across $\rho$ values we combine *P* values using ACAT.

For ACATV, we first compute the marginal association *P* values ${p}_{j}$ for each variant *j* from the score statistics ${S}_{j}$ and their variance. Then the *P* values are combined using the test statistic

$${Q}_{{\rm{A}}{\rm{C}}{\rm{A}}{\rm{T}}-{\rm{V}}}=\mathop{\sum }\limits_{j=1}^{p} \bar{w}_{j}\tan ((0.5-{p}_{j})\pi )$$

$${\bar{w}}_{j}=\frac{{w}_{j}^{2}\,{f}_{j}(1-{f}_{j})}{{\sum }_{j{\prime} }{w}_{{j}^{{\prime} }}^{2}{f}_{{j}^{{\prime} }}(1-{f}_{{j}^{{\prime} }})}$$

${Q}_{{\rm{A}}{\rm{C}}{\rm{A}}{\rm{T}}-{\rm{V}}}$ follows a standard Cauchy distribution.

REMETA, like MetaSTAAR, combines *P* values across gene-based tests into a single omnibus test termed ‘GENE_ P.’ GENE P *P* values are computed in two steps. In the first step, *P* values are combined per test using ACAT into BURDEN-ACAT, ACATV-ACAT and SKATO-ACAT *P* values. In the second step, per test *P* values are combined with ACAT to compute GENE P.

### Approximate gene-based testing using per study LD

The matrix Cov(**S**) needs to be computed and stored for every trait and every study in the analysis. We therefore wanted to approximate Cov(**S**) with a matrix computed once per study. As others have noted^15,19^, in the absence of covariates (except an intercept) ${\rm{C}}{\rm{o}}{\rm{v}}(\bf{S})=\nu {\rm{C}}{\rm{o}}{\rm{v}}(G^T)$ where *ν* is a scalar that depends on the phenotype. This suggests we should compute ${\rm{Cov}}(G^T)$ once, then adjust it for the phenotype being testing. A natural choice is to adjust ${\rm{Cov}}(G^T)$ to have the same diagonal as Cov(**S**). This is the approach we take.

Let *G* be the *n* *×* *p* genotype matrix of all variants in a gene, $\bf{S}_{t}$ be the score statistics of all variants in a gene for a particular trait *t* and ${\Phi }_{t}={\rm{Cov}}(\bf{S}_{t})$ be their covariance. We propose storing three pieces of information to construct gene-based tests: $\bf{S}_{t}$, $D_t=\mathrm{diag}\left({\Phi }_{t}\right)$ and $\mathrm{Cov}({{G}}^{{\rm{T}}})$. We then approximate (note that ${\rm{Corr}}({G}^{T})$ is easy to compute from ${\rm{Cov}}({G}^{T})$):

$${\rm{Cov}}\left({{\bf{S}}}_{t}\right)\approx {D_t}^{\frac{1}{2}}{\rm{Corr}}\left({{\rm{G}}}^{T}\right){D_t}^{\frac{1}{2}}$$

Thus, for *T* traits the time and space requirements are reduced from ${\mathscr{O}}{\mathscr{(}}T{p}^{2}+{Tp})$ (*T* matrices of size *p* *×* *p* plus the score statistics) to ${{\mathscr{O}}{\mathscr{(}}p}^{2}+2{Tp})$ (one matrix of size *p* *×* *p*, the score statistics, and the diagonal of ${\Phi }_{t}$).

### Effect size meta-analysis

Effect sizes are meta-analyzed using an inverse-variance weighted meta-analysis. Specifically, if ${\beta }_{1},\ldots ,{\beta }_{K}$ and ${{SE}}_{1},\ldots ,{{SE}}_{K}$ are the effect sizes and s.e. values for a variant across *K* studies, we compute

$${w}_{k}=\frac{1}{{\left(s.e{.}_{k}\right)}^{2}}$$

$$\beta =\frac{{\sum }_{k=1}^{K}{w}_{k}{\beta }_{k}}{{\sum }_{k=1}^{K}{w}_{k}}$$

$${\rm{s}}.{\rm{e}}.={\left(\frac{1}{{\sum }_{k=1}^{K}{w}_{k}}\right)}^{\frac{1}{2}}$$

$${{Q}_{{\rm{E}}{\rm{S}}{\rm{M}}{\rm{A}}}=\left(\frac{\beta }{{\rm{s}}.{\rm{e}}.}\right)}^{2}\sim {\chi }^{2}(1)$$

### *P* value meta-analysis

PVMA is performed using Stouffer’s method. In Stouffer’s method, *P* values are first converted to *z*-scores, then combined by taking a sum weighted by the sample sizes of each study. Let ${p}_{1},\ldots ,{p}_{K}$ be the *P* values for a test across *K* studies with sample sizes ${N}_{1},\ldots ,{N}_{K}$. We compute

$${Z}_{k}={\Phi }^{-1}\left({p}_{k}\right)$$

$${w}_{k}=\sqrt{{N}_{k}}$$

$$Z=\frac{{\sum }_{k}{{w}_{k}Z}_{k}}{\sqrt{{\sum }_{k}{w}_{k}^{2}}}\sim N(0,1)$$

### Meta-analysis of gene-based tests

Let ${S}_{k}$ be the *j* *×* 1 vector of score statistics for variants in a gene-based test in study *k*. For meta-analysis of WST and SKATO across studies, we can combine the score statistics across studies

$$\bf{S}=\mathop{\sum }\limits_{k=1}^{K}\bf{{S}}_{k}$$

$$\mathrm{Cov}\left(\bf{S}\right)=\mathop{\sum }\limits_{k=1}^{K}\mathrm{Cov}(\bf{S}_{k})$$

Then *P* values can be computed as in the single study case above.

### Estimation of effect sizes

In the sum test, each variant in a mask is assumed to have the same effect size.

Consequently, if *β* is the true effect size to be estimated, and ${\hat{\beta }}_{1},\ldots ,{\hat{\beta }}_{p}$ are the marginal effect size estimates of the variants in the mask, then ${\mathbb{E}}{\mathbb{[}}{\hat{\beta }}_{j}]=\beta$ for each variant *j* in the mask. Furthermore, for any convex combination ${w}_{1},\ldots ,{w}_{p}$ of the effect sizes we have ${\mathbb{E}}\left[{\sum }_{j}{w}_{j}{\hat{\beta }}_{j}\right]={\sum }_{j}{w}_{j}{\mathbb{E}}\left[{\hat{\beta }}_{j}\right]=\beta$. We could use this observation to look for an estimator that minimizes ${\rm{Var}}\left({\sum }_{j}{w}_{j}{\hat{\beta }}_{j}\right)$. If there is no correlation between the effect sizes in the mask, this is equivalent to an inverse-variance weighted meta-analysis of the variants in the mask. We use the s.e. $s=\sqrt{{\rm{Var}}\left({\sum }_{j}{w}_{j}{\hat{\beta }}_{j}\right)}$ of this estimate to rescale the *z*-score of the sum test. Specifically, if *z* is the *z*-score of the sum test, then the estimated effect size is $\hat{\beta }={sz}$. In practice, we found that settings the weights to ${w}_{j}\propto \frac{1}{{({{\rm{s}}.{\rm{e}}.}_{{\rm{j}}})}^{2}}$, the weights used by inverse-variance meta-analysis, produces accurate estimates of effect sizes.

For meta-analysis of burden tests, we first estimate effect sizes and standard errors per study. Then we combine effect sizes and s.e. across studies using inverse-variance weighted meta-analysis.

### Conditional analysis

REMETA stores the LD of variants within a gene and in a user-specified buffer region around each gene. As long as a variant is stored in the LD matrix of a gene it can be used to perform conditional analysis of gene-based tests. If we let $\bf{S}_{{\rm{g}}}$ and $\bf{S}_{{\rm{c}}}$ be the score statistics in a gene-based test and the score statistics of variants to condition on, then, under the null hypothesis

$$\bf{S}_{{\rm{g}}}|\bf{S}_{{\rm{c}}}=\bf{S}_{{\rm{g}}}-{\rm{C}}{\rm{o}}{\rm{v}}(\bf{S}_{{\rm{g}}},\bf{S}_{{\rm{c}}}){\rm{C}}{\rm{o}}{\rm{v}}{(\bf{S}_{{\rm{c}}})}^{-1}\bf{S}_{{\rm{c}}}$$

$${\rm{C}}{\rm{o}}{\rm{v}}(\bf{S}_{{\rm{g}}}|\bf{S}_{{\rm{c}}})={\rm{C}}{\rm{o}}{\rm{v}}(\bf{S}_{{\rm{g}}})-{\rm{C}}{\rm{o}}{\rm{v}}(\bf{S}_{{\rm{g}}},\bf{S}_{{\rm{c}}}){\rm{C}}{\rm{o}}{\rm{v}}{(\bf{S}_{{\rm{c}}})}^{-1}{\rm{C}}{\rm{o}}{\rm{v}}{(\bf{S}_{{\rm{g}}},\bf{S}_{{\rm{c}}})}^{T}$$

For meta-analysis, we perform conditional analysis across all studies at once, that is, on the sum of score statistics across studies.

We also estimate the conditional effect sizes of gene-based tests. If $\boldsymbol{\beta }_{{\rm{g}}}$ and $\boldsymbol{\beta }_{{\rm{c}}}$ are the effect size estimates for the variants in a gene-based test and effect size estimates of the variants to condition on, then we compute

$$\boldsymbol{\beta }_{{\rm{g}}}|\boldsymbol{\beta }_{{\rm{c}}}=\boldsymbol{\beta }_{{\rm{g}}}-{\rm{C}}{\rm{o}}{\rm{v}}(\boldsymbol{\beta }_{{\rm{g}}},\boldsymbol{\beta }_{{\rm{c}}}){\rm{C}}{\rm{o}}{\rm{v}}{(\boldsymbol{\beta }_{{\rm{c}}})}^{-1}\boldsymbol{\beta }_{{\rm{c}}}$$

$${\rm{C}}{\rm{o}}{\rm{v}}(\boldsymbol{\beta }_{{\rm{g}}}|\boldsymbol{\beta }_{{\rm{c}}})={\rm{C}}{\rm{o}}{\rm{v}}(\boldsymbol{\beta }_{{\rm{g}}})-{\rm{C}}{\rm{o}}{\rm{v}}(\boldsymbol{\beta }_{{\rm{g}}},\boldsymbol{\beta }_{{\rm{c}}}){\rm{C}}{\rm{o}}{\rm{v}}{(\boldsymbol{\beta }_{{\rm{c}}})}^{-1}{\rm{C}}{\rm{o}}{\rm{v}}{(\boldsymbol{\beta }_{{\rm{g}}},\boldsymbol{\beta }_{{\rm{c}}})}^{T}$$

We estimate the covariance of $$\boldsymbol{\beta} :={[\begin{array}{cc}\boldsymbol{\beta }_{{\rm{g}}}^{T} & \boldsymbol{\beta }_{{\rm{c}}}^{T}\end{array}]}^{T}$$ using a similar strategy to the approximation for score statistics. If $D={\rm{diag}}\left\{{\rm{Cov}}\left( \boldsymbol{\beta} \right)\right\}$, then we approximate ${\rm{Cov}}\left( \boldsymbol{\beta} \right)={D}^{\frac{1}{2}}{\rm{Corr}}\left({G}^{T}\right){D}^{\frac{1}{2}}$ where *G* is the *n* *×* *p* matrix of variants in the test. This is similar to the strategy used by COJO^26^.

For meta-analysis, we first condition the effect sizes within each study, then combine them using the method described in ‘Estimation of effect sizes.’

### Estimation of genotype counts and allele frequencies

If genotype counts of the single variants are known, then we can use them to estimate genotype counts of the burden mask. Let ${{\bf{G}}}_{1},\ldots ,{{\bf{G}}}_{p}$ be the vectors of genotypes at the *p* variants in the mask, and let ${\bf{Y}}=max\{{{\bf{G}}}_{1},\ldots ,{{\bf{G}}}_{p}\}\in \{0,1,2\}$ be the mask genotype vector calculated by taking the maximum genotype across elements of the variant vectors. Additionally, let ${N}_{{G}_{j}=1}$ be the number of individuals who are heterozygotes at variant *j*, and ${N}_{Y=1}$ be the number of individuals who are heterozygous for the burden mask. We want to estimate ${N}_{Y=1}$ from ${N}_{{G}_{1}=1},\ldots ,{N}_{{G}_{p}=1}$. Specifically, we want to find coefficients ${c}_{j}$ such that

$${N}_{Y=1}=\mathop{\sum }\limits_{j}{c}_{j}{N}_{{G}_{j}=1}$$

The strategy we take is to compute ${c}_{j}$ sequentially, such that each ${c}_{j}$ estimates the proportion of the ${N}_{{G}_{j}=1}$ we have yet to count. Specifically,

$${c}_{j}=\Pr \left({G}_{1}\ne 1,\ldots ,{G}_{j-1}\ne 1,|,{G}_{j}=1\right)\approx \mathop{\prod }\limits_{m=1}^{j-1}\Pr \left({G}_{m}\ne 1,|,{G}_{j}=1\right)$$

The approximation is chosen because the terms $\Pr ({G}_{m}\ne 1|{G}_{j+1}=1)$ can be computed from the LD matrix (the case for homozygotes is similar).

### Extension to binary traits with case–control imbalance

For unbalanced binary traits, using a normal approximation to the distribution of the score statistic can lead to inflated type I error. SPA has been shown to be an effective strategy for controlling type I error for both single variants^27^ and gene-based tests^28^. SPA uses the cumulant generating function of the score statistic to approximate its null distribution. For a variant *j*, the cumulant generating function of the score statistic for logistic regression is

$$K\left(t\right)=\mathop{\sum }\limits_{i=1}^{n}\log \left({{\mathbb{E}}}_{{H}_{0}}\left[{e}^{t{S}_{j}}\right]\right)$$

$$=\mathop{\sum }\limits_{i=1}^{n}\log \left(1-{\hat{\mu }}_{i}+{\hat{\mu }}_{i}{e}^{{g}_{{ij}}t}\right)-t\mathop{\sum }\limits_{i=1}^{n}{g}_{{ij}}{\hat{\mu }}_{i}$$

where ${\hat{\mu }}_{i}$ is estimated under the null hypothesis. In the setting of single-variant meta-analysis, SPA has been extended a summary statistics approach by recognizing that, if the model only includes an intercept, then an SPA can be computed from case–control counts and genotype counts alone^24^. Briefly, for a study *s* let ${n}_{{\rm{c}}{\rm{a}}{\rm{s}}{\rm{e}}{\rm{s}}}^{s},{n}_{{\rm{c}}{\rm{o}}{\rm{n}}{\rm{t}}{\rm{r}}{\rm{o}}{\rm{l}}{\rm{s}}}^{s}$ be the number of case and number of controls. Let ${n}_{\rm{homref}}^{s},{n}_{\rm{het}}^{s},{n}_{\rm{homalt}}^{s}$ be the number of homozygous reference, heterozygous and homozygous alternate genotypes for a mask. Then we have

$$\hat{\mu }=\frac{{\sum }_{s}{n}_{{\rm{c}}{\rm{a}}{\rm{s}}{\rm{e}}{\rm{s}}}^{s}}{{\sum }_{s}{n}_{{\rm{c}}{\rm{a}}{\rm{s}}{\rm{e}}{\rm{s}}}^{s}+{n}_{{\rm{c}}{\rm{o}}{\rm{n}}{\rm{t}}{\rm{r}}{\rm{o}}{\rm{l}}{\rm{s}}}^{s}}$$

$${n}_{x}=\mathop{\sum }\limits_{s}{n}_{x}^{s},x\in \{\mathrm{homref},\mathrm{het},\mathrm{homalt}\}$$

The cumulant generating function for the SPA is

$$K(t)={\sum}_{x\in \left\{{\rm{homref}},{\rm{het}},{\rm{homalt}}\right.}{n}_{x}{\rm{l}}{\rm{o}}{\rm{g}}(1-{\hat{\mu}}+{\hat{\mu}}{e}^{{g}_{x}t})$$

where ${g}_{x}$ is the mean centered genotype divided by $\sqrt{{\rm{Var}}(S_j)}$.

REMETA computes two types of SPAs: a mask-based SPA and a variant-based SPA. For the mask-based SPA, we use the observation that the sum test approximates the collapsing variant test, and that we can we estimate genotype counts of collapsing variant test. This allows us to compute a *P* value for the sum test, and use that *P* value to compute a calibration factor for burden tests and SKATO in a similar strategy to that in ref. ^29^. Let ${p}_{{\rm{S}}{\rm{T}}}$ and ${p}_{{\rm{S}}{\rm{P}}{\rm{A}}}$ be the *P* values computed from the sum test and SPA respectively. Let $r=max(1,\frac{{\chi }_{{\rm{q}}{\rm{u}}{\rm{a}}{\rm{n}}{\rm{t}}{\rm{i}}{\rm{l}}{\rm{e}}}^{2}(1-{p}_{{\rm{S}}{\rm{T}}})}{{\chi }_{{\rm{q}}{\rm{u}}{\rm{a}}{\rm{n}}{\rm{t}}{\rm{i}}{\rm{l}}{\rm{e}}}^{2}(1-{p}_{{\rm{S}}{\rm{P}}{\rm{A}}})})$, then we replace the matrix ${\rm{Cov}}(\bf{S})$ by $r{\rm{Cov}}(\bf{S})$.

For the variant-based SPA, we compute a similar calibration factor for the variance of the score statistics. Let *P*_NM_ and *P*_SPA_ be the *P* values computed by a normal approximation (the standard approach) and the SPA for a single variant $j$. Let ${r}_{j}=max(1,\frac{{\chi }_{{\rm{q}}{\rm{u}}{\rm{a}}{\rm{n}}{\rm{t}}{\rm{i}}{\rm{l}}{\rm{e}}}^{2}(1-{p}_{{\rm{N}}{\rm{M}}})}{{\chi }_{{\rm{q}}{\rm{u}}{\rm{a}}{\rm{n}}{\rm{t}}{\rm{i}}{\rm{l}}{\rm{e}}}^{2}(1-{p}_{{\rm{S}}{\rm{P}}{\rm{A}}})})$, then we replace the variance ${\rm{Var}}({S}_{j})$ by ${r}_{j}{\rm{Var}}({S}_{j})$.

### Variant annotations and gene sets

We grouped variants into sets (or masks) for gene-based tests based on variant annotations and allele frequencies. Variants were grouped based on seven annotation categories: predicted loss of function (pLoF) variants, deleterious missense variants, possibly deleterious missense variants, all missense variants and pLoFs combined with each missense category. Variants were annotated using VEP^12^ using the variant from canonical transcripts. Variants annotated with stop gained, start lost, splice donor, splice acceptor, stop lost or frameshift were considered pLoFs. For missense variants, results from five prediction algorithms were used to determine their severity: SIFT^30^, PolyPhen2 HDIV, PolyPhen2 HVAR^31^, LRT^32^ and MutationTaster^33^. Variants were grouped into a deleterious missense category if predicted deleterious by all five algorithms, a possibly deleterious category if predicted deleterious by at least one algorithm and an all missense category if not predicted deleterious by any algorithm. For comparisons between REGENIE and REMETA, we considered four allele frequency bins: AAF < 1%, AAF < 0.1%, AAF < 0.001% and singletons. For comparisons between standard meta-analysis and REMETA, we considered five allele frequency bins: AAF < 1%, AAF < 0.5%, AAF < 0.1%, AAF < 0.001% and singletons.

For the BURDEN-ACAT test, we aggregated the *P* values from the WST applied to all groups at all AAF bins. For the SKATO-ACAT and ACATV-ACAT tests we aggregated the *P* values from SKATO and ACATV tests applied to all groups using a single AAF cutoff of 1% along with minor allele frequency-dependent variant weights. In each case aggregation was applied using ACAT (Cauchy combination method). The BURDEN-ACAT, SKATO-ACAT and ACATV-ACAT tests were then aggregated into a single GENE_P^21^ *P* value using ACAT.

### Analysis and meta-analysis of UK Biobank

We analyzed WES data from the final release of the OQFE pipeline along with imputed genotypes for 469,376 samples in UKB. Details of the exome sequencing^34^, phenotyping and array genotyping^35^, and imputation against TOPMed and ancestry assignment^1^, have been described previously. Both BMI and LDL were transformed using the rank-based inverse-normal approach. Association testing in REGENIE was performed using the covariates age, age^2^, sex, age by sex, exome batch, the top ten array PCs and the top 20 exome PCs.

### Description of ESM-1v annotation methods

In the meta-analysis of UKB, we tested two additional masks using ESM-1v annotations to test for associations: ESM-1v deleterious missense mutations predicted by the ESM-1v model and ESM-1v deleterious missense mutations combined with pLoFs. We downloaded the ESM-1v model from https://github.com/facebookresearch/esm. We computed the wildtype marginal score averaged across all five ESM-1v models. For proteins with sequences longer than 1,022 amino acids, we centered the variant in the 1,022 amino sequence. In cases where the variant was near the end of the protein, we included the maximal amount of variant amino acid context. Variants in the top 22% of missense scores were grouped into a ‘deleterious missense’ category. We chose a 22% cutoff so that the same number of variants would be included in the deleterious missense category defined by the five prediction algorithms above as the ESM-1v predictions.

### Reporting summary

Further information on research design is available in the Nature Portfolio Reporting Summary linked to this article.

## Online content

Any methods, additional references, Nature Portfolio reporting summaries, source data, extended data, supplementary information, acknowledgements, peer review information; details of author contributions and competing interests; and statements of data and code availability are available at 10.1038/s41588-025-02390-0.

## Supplementary information

Supplementary Information Supplementary Note 1, Figs. 1–15 and List of investigators from the Regeneron Genetics Center.

Reporting Summary

Supplementary Table 1 Significant gene–trait associations in meta-analysis of three equal subsets of UKB. Gene–trait pairs are included if any of the GENE_P, BURDEN-ACAT, ACATV-ACAT or SKATO-ACAT *P* values are ExWAS significant (*P* < 0.05/20,000).

## Figures and Tables

**Extended Data Table 1:**
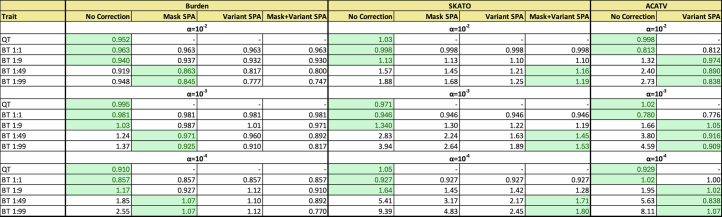
Evaluation of type 1 error rates for gene-based tests

Each cell is empirical type 1 error divided by the significance level α averaged across 10 simulation replicates. Null traits were simulated across three equally sized subsets of UK Biobank and meta-analyzed with REMETA. Gene-sets were constructed across three annotations categories—pLoFs, all missense variants, and synonymous variants—using an allele frequency cutoff of 1%. Overall, 57,165 gene-based tests were performed for each trait. Cells in green show type 1 error rates using REMETA’s default parameters.

## Data Availability

UKB phenotype data, genotyping array data and WES data can be accessed through the UKB research analysis platform (https://ukbiobank.dnanexus.com/landing). Significant gene–trait associations from the meta-analysis of UKB are available in Supplementary Table 1.

## Code availability

The C++ source code and precompiled binaries for REMETA v.0.9.0 are available from https://rgcgithub.github.io/remeta/ and via Zenodo at 10.5281/zenodo.17136106 (ref. ^18^). REMETA is distributed under an MIT License. Source code a precompiled for binaries for REGENIE v.3.4.1 are available from https://rgcgithub.github.io/regenie/. Source code for metaSTAAR v.0.9.6.3 is available from https://github.com/xihaoli/MetaSTAAR.
