# Supplementary material for: Computationally efficient meta-analysis of gene-based tests using summary statistics in large-scale genetic studies
Source: Nat Genet. 2025 Nov 12;57(12):3193–200. doi: 10.1038/s41588-025-02390-0 (PMC12695653; doi:10.1038/s41588-025-02390-0)
Supplement: Supplementary file 1 — Supplementary Note 1, Figs. 1–15 and List of investigators from the Regeneron Genetics Center. [file 41588_2025_2390_MOESM1_ESM.pdf]

nature genetics

Supplementary information https://doi.org/10.1038/s41588-025-02390-0

Computationally efficient meta-analysis of gene-based tests using summary statistics in large-scale genetic studies

In the format provided by the authors and unedited

Supplementary Information for "Computationally efficient meta-analysis of gene-based tests using summary statistics in large-scale genetic studies"

August 12, 2025

Contents

1 Supplementary Note: Details of gene-based tests **2**
1.1 Burden testing . . . 2
1.1.1 Estimating effect sizes . . . 3
1.2 The sequence kernel association test (SKAT) . . . 3
1.2.1 Computing the null distribution of Q_{SKAT} . . . 4
1.2.2 Extension to SKATO . . . 5
1.2.3 Computing the score statistic for a quantitative trait . . . 5
1.2.4 Computing the score statistic for a binary trait . . . 6
1.3 Aggregated Cauchy association test (ACATV) . . . 7
1.4 Saddlepoint approximation for unbalanced binary traits . . . 7
1.5 Estimating mask genotype counts and allele frequencies . . . 8
1.5.1 Preliminaries . . . 8
1.5.2 Genotype counts . . . 9
1.5.3 Estimating allele frequencies when genotype counts are unavailable . . . 10

2 Supplementary Figures **12**

3 List of investigators from the Regeneron Genetics Center **27**

1 Supplementary Note: Details of gene-based tests

Here we provide additional mathematical details of the gene-based tests implemented in REMETA. For each test the set up is as follows. Suppose we have phenotypes y_i for $i = 1...n$ samples. Let $x_i \in \mathbb{R}^m$ be a vector of covariates (including the intercept) per sample and $X \in \mathbb{R}^{n \times m}$ a matrix with rows x_i^T. Let $g_i \in \{0, 1, 2\}^p$ be the vector counting the number of alternate alleles at each variant in a test and $G \in \mathbb{R}^{n \times p}$ a matrix with rows g_i^T. To keep notation consistent across sections individuals will be indexed from $i = 1...n$ and variants from $j = 1...p$.

1.1 Burden testing

Our goal for burden testing in REMETA is to approximate meta-analysis of the default burden test in REGENIE, which uses collapsing variant test [1]. The collapsing variant test assumes that multiple damaging variants in the gene have the same impact as having just one. All the genotypes in the mask get collapsed into a single composite genotype:

$$C_i = \max\{g_{i1}, ..., g_{ip}\}$$

The max operator makes it challenging to reconstruct the collapsing burden test from single variant summary statistics. Instead, burden testing in REMETA is performed using a variation of the weighted sum test (WST) [2]. The WST takes a weighted sum of the variants in a mask to construct a composite genotype

$$C_i = \sum_{j=1}^{p} w_j g_{ij}$$

The w_j are pre-specified variant weights. Here, each damaging variant in the mask is assumed to increases the magnitude of the effect on the phenotype. That effect can be weighted by different characteristics of the variant like its allele frequency. If the weights $w_j = 1$, and an individual has at most one damaging variant in the mask, then the collapsing variant test is equivalent to the WST. We refer to the special case of the WST with $w_j = 1$ as the sum test.

As most individuals are unlikely to have multiple rare damaging mutations in a single gene, we expect the composite genotypes for the collapsing variant test and the sum test to be similar. Indeed, p-values in from the sum test approximate p-values from the collapsing burden test (Supplementary Figure 4).

For both models association testing is performed using linear regression for quantitative traits and logistic regression for binary traits. For quantitative traits the model is

$$\begin{aligned} \eta_i &= x_i^T \alpha + C_i \beta \\ \mu_i &= \eta_i \\ y_i &\sim \mathcal{N}(\mu_i, \sigma^2) \end{aligned}$$

where σ^2 is the residual variance. For binary traits the model is

$$\begin{aligned} \eta_i &= x_i^T \alpha + C_i \beta \\ \mu_i &= \text{logistic}(\eta_i) \\ y_i &\sim \text{Bernoulli}(\mu_i) \end{aligned}$$

The benefit of the WST compared to the collapsing variant test is that a score test can be constructed from the score statistics of the individual variants and their covariance [3]

$$\begin{aligned} S &= G^T (y - \hat{\mu}) \\ \text{Cov}(S) &= G^T \text{Cov}(y - \hat{\mu}) G \end{aligned}$$

where $\hat{\mu}$ is estimated under the null hypothesis using either linear regression or logistic regression. The score statistic of the WST is given by $w^T S$, so we have

$$\begin{aligned} Q_{WST} &= (w^T S)^2 \\ \frac{Q_{WST}}{w^T \text{Cov}(S) w} &\sim \chi^2(1) \end{aligned}$$

1.1.1 Estimating effect sizes

To estimate an effect size for the sum test, we use the observation that in the sum test each variant is assumed to have the same effect size. Specifically, if $\beta_1, ..., \beta_p$ are the effect sizes for the p variants in a mask, then the sum test assumes $\beta := \beta_1 = \beta_2 = \cdots = \beta_p$. Thus, if $\hat{\beta}_1, ..., \hat{\beta}_p$ are the marginal estimates, then $\mathbb{E}[\hat{\beta}_j] = \beta$ for each variant j in the mask. In principle, we could seek a convex combination $w_1, .., w_p$ of the effect sizes to construct and estimator for β. Let $\hat{\beta} = (\hat{\beta}_1, ...\hat{\beta}_j)^T$ and $w = (w_1, ..., w_p)^T$. Then we want to find

$$\begin{aligned} &\min_w \operatorname{Cov}(w^T \hat{\beta}) \\ \text{s.t.} \quad & w_j \geq 0 \text{ for } j = 1...p \\ &\sum_j w_j = 1 \end{aligned}$$

That is, we want to find a minimum variance unbiased estimator for β. If there is no correlation among the $\hat{\beta}$, then solution is equivalent to an inverse-variance weighted meta-analysis.

We can solve for the optimal weights using the method of Lagrange multipliers. The Lagrangian is

$$\mathcal{L}(w, \lambda) = w^T \Sigma w - \lambda(w^T \mathbf{1} - 1)$$

Taking derivatives with respect to w and λ and setting them to zero gives us

$$\begin{aligned} \nabla_w \mathcal{L}(w, \lambda) &= 2\Sigma w - \lambda \mathbf{1} = 0 \\ \implies w &= \frac{\lambda}{2} \Sigma^{-1} \mathbf{1} = \lambda v \\ v &:= \frac{1}{2} \Sigma^{-1} \end{aligned}$$

Since

$$1 = \sum_k w_k = \lambda \sum_k v_k$$

we have

$$\lambda = \frac{1}{\sum_k v_k} = \frac{1}{\frac{1}{2} \mathbf{1}^T \Sigma^{-1} \mathbf{1}}$$

Then the optimal weights are [4]

$$w = \frac{\Sigma^{-1} \mathbf{1}}{\mathbf{1}^T \Sigma^{-1} \mathbf{1}}$$

Using the optimal weights would require inverting Σ per mask, which can be computationally expensive. In practice, we using the weights from an inverse-variance meta-analysis (i.e. $\Sigma = \operatorname{diag}\{\operatorname{Var}(\hat{\beta}_1), ..., \operatorname{Var}(\hat{\beta}_p)\}$). performs well.

In order to ensure the estimated effect size is consistent with the test statistic of the sum test, we use the standard error of the effect size estimate to rescale the z-score of the sum test. The standard error is given by $s = \sqrt{\operatorname{Cov}(w^T \hat{\beta})}$. Thus, if z is the z score from the sum test, the estimated effect size is $\hat{\beta} = sz$. The standard error is then recomputed from the p-value of the sum test.

1.2 The sequence kernel association test (SKAT)

Here we present a derivation for a special case of the SKAT and SKATO tests where the effect sizes follow a normal distribution. SKAT uses a variance component model where the effect sizes β_j are the random effects. Let $\beta \in \mathbb{R}^p$, then the model we consider is

$$\begin{aligned} \beta &\sim N(0, \tau W) \\ W &: = \operatorname{diag}(w_1, ..., w_p) \end{aligned}$$

The w_j are fixed weight parameters and τ is the variance component. For quantitative traits the phenotype is related to the genotypes by

$$\mu_i = x_i^T \alpha + g_i^T \beta$$
$$y_i | \beta \sim N\left(\mu_i, \sigma^2\right)$$

where α are the fixed effects. For binary traits the SKAT model is

$$\text{logit}(\mu_i) = x_i^T \alpha + g_i^T \beta$$
$$y_i | \beta \sim \text{Bernoulli}(\mu_i)$$

SKAT tests the null hypothesis $\tau = 0$ against the alternate hypothesis $\tau > 0$. We show in section 1.2.3 for quantitative traits and section 1.2.4 for binary traits that the test statistic for a score test is given by

$$Q_{SKAT} = (y - \hat{\mu})^T G W G^T (y - \hat{\mu})$$

where $\hat{\mu}$ is mean under the null hypothesis ($\tau = 0$).

1.2.1 Computing the null distribution of Q_{SKAT}

We want to compute the null distribution of Q_{SKAT}. The terms $S := G^T(y - \hat{\mu})$ are the score statistics of the individual variants conditional on the estimate of α. To compute their variance, note that in the limit of a large sample

$$\begin{bmatrix} G^T(y-\mu) \\ X^T(y-\mu) \end{bmatrix} \sim \mathcal{N}\left(\begin{bmatrix} 0 \\ 0 \end{bmatrix}, \begin{bmatrix} G^T V G & G^T V X \\ X^T V G & X^T V X \end{bmatrix} \right)$$

where $V = \sigma^2 I$ for quantitative traits and $V = \text{diag}\{\mu_1(1-\mu_1), ..., \mu_n(1-\mu_n)\}$ for binary traits. At the maximum likelihood estimate of α we have $X^T(y - \mu) = 0$, so

$$\begin{aligned} G^T(y-\mu) | X^T(y-\mu) &\sim \mathcal{N}(0, G^T V G - G^T V X (X^T V X)^{-1} X^T V G) \\ &= \mathcal{N}(0, G^T P G) \\ P :&= V - VX(X^T V X)^{-1} X^T V \end{aligned}$$

Thus, under the null hypothesis ($\tau = 0$) we have

$$\begin{aligned} G^T(y - \hat{\mu}) &\sim \mathcal{N}\left(0, G^T P_0 G\right) \\ P_0 &:= V - VX(X^T V X)^{-1} X^T V \end{aligned}$$

where $\hat{\mu}$ and P_0 is estimated under the null.

Now consider $\text{Var}(W^{1/2}S) = \text{Var}(W^{1/2}G^T(y - \hat{\mu})) = W^{1/2}G^T P_0 G W^{1/2}$. The matrix is positive semi-definite, so it has an eigendecomposition $\Sigma\Lambda\Sigma^T$. Let

$$z = \Lambda^{-1/2}\Sigma^T \left(W^{1/2} G^T (y - \hat{\mu}) \right)$$

and note that z is $\mathcal{N}(0, I)$. Then we can write Q_{SKAT} in terms of z

$$\begin{aligned} Q_{SKAT} &= (y - \hat{\mu})^T G W G^T (y - \hat{\mu}) \\ &= z^T \Lambda^{1/2} \Sigma^T \Sigma \Lambda^{1/2} z \\ &= z \Lambda z \\ &= \sum_{j=1}^{p} \lambda_j z_j^2 \end{aligned}$$

Each z_j^2 is a $\chi^2(1)$ random variable. The λ_j are the eigenvalues of $W^{1/2}G^T P_0 G W^{1/2}$. Thus Q_{SKAT} has a mixture of chi-square distribution.

1.2.2 Extension to SKATO

The SKAT model assumes the random effects β_j are uncorrelated. If we instead test a model where the β_j are correlated we get the model used by SKATO:

$$\begin{aligned}\beta &\sim N\left(0, \tau W^{1/2}\left((1-\rho)I + \rho\mathbf{1}\mathbf{1}^T\right)\right) W^{1/2}\Big) \\ W &= \text{diag}(w_1, .., w_p)\end{aligned}$$

The term ρ is the correlation between each pair of β_k and $\beta_{k'}$. Let $R_\rho = (1-\rho)I + \rho\mathbf{1}\mathbf{1}^T$. Replacing τW by $\tau W^{1/2} R_\rho W^{1/2}$ in the derivations in sections 1.2.3 and 1.2.4 gives us the score statistic for the variance component test

$$Q_\rho = (y - \hat{\mu})^T G W^{1/2} R_\rho W^{1/2} G^T (y - \hat{\mu})$$

We can expand Q_ρ into a weighted sum of a burden test and Q_{SKAT}

$$\begin{aligned}Q_\rho &= (y-\hat{\mu})^T G W^{1/2}((1-\rho)I + \rho\mathbf{1}\mathbf{1}^T)W^{1/2}G^T(y-\hat{\mu}) \\ &= (1-\rho)(y-\hat{\mu})^T G W^{1/2} W^{1/2} G^T (y-\hat{\mu}) + \rho(y-\hat{\mu}) G W^{1/2}\mathbf{1}\mathbf{1}^T W^{1/2} G^T (y-\hat{\mu}) \\ &= (1-\rho)\sum_{j=1}^p \left(w_j^{1/2}\sum_{i=1}^n g_{ij}(y_i - \hat{\mu}_i)\right)^2 + \rho\left(\sum_{j=1}^p w_j^{1/2}\sum_{i=1}^n g_{ij}(y_i-\hat{\mu}_i)\right)^2 \\ &= (1-\rho)\left(\sum_{j=1}^p w_j S_j^2\right) + \rho\left(\sum_{j=1}^p w_j^{1/2} S_j\right)^2 \\ &= (1-\rho)Q_{SKAT} + \rho Q_{BURDEN}\end{aligned}$$

1.2.3 Computing the score statistic for a quantitative trait

The score statistic is defined to be the derivative of the log likelihood. Thus we first need to compute

$$L(\alpha, \tau) := p(y) = \int p(y|\beta)p(\beta)\, d\beta = \int N(y|X\alpha + G\beta, \sigma^2 I)N(\beta|0, \tau W)\, d\beta$$

The computation for marginalizing a product of Gaussians is well known (e.g. see [5]) The result is

$$p(y) = N\left(y|X\beta, \sigma^2 I + \tau G W G^T\right)$$

The log-likelihood of y is

$$l(\alpha,\tau) = -\frac{1}{2}(y - X\alpha)^T\left(\sigma^2 I + \tau GWG^T\right)^{-1}(y - X\alpha) - \frac{1}{2}\log\left|\sigma^2 I + \tau GWG^T\right| + \text{const wrt } \tau$$

Taking derivatives with respect to τ gives us the score statistic. For the first term write out the quadratic form as a sum, use $\frac{\partial}{\partial x}A^{-1} = -A^{-1}(\frac{\partial}{\partial x}A)A^{-1}$, then rewrite as a quadratic form. For the second term use $\frac{\partial}{\partial x}\log|A| = \text{Tr}(A^{-1}(\frac{\partial A}{\partial x}))$.

$$\begin{aligned}\frac{\partial l}{\partial \tau} = &\frac{1}{2}(y-X\alpha)^T(\sigma^2 I + \tau GWG^T)^{-1}GWG^T(\sigma^2 I + \tau GWG^T)^{-1}(y - X\alpha) \\ &- \frac{1}{2}\text{Tr}\left(\left(\sigma^2 I + \tau GWG^T\right)^{-1} GWG^T\right)\end{aligned}$$

Under the null hypothesis $H_0 : \tau = 0$ we have

$$\frac{1}{2\sigma^4}(y - X\alpha)^T GWG^T(y - X\alpha) - \frac{1}{2}\text{Tr}\left((\sigma^2 I)^{-1} + GWG^T\right)$$

Note that only the first term is random while the second term is constant. Therefore to test the null hypothesis we only need the first term. Rescaling by $2\sigma^4$ gives us the SKAT test statistic

$$Q = (y - \mu)^T GWG^T(y - \mu)$$

1.2.4 Computing the score statistic for a binary trait

Similar to above, we want to compute

$$L(\alpha, \tau) := p(y) = \int p(y|\beta)p(\beta)\, d\beta = \int \prod_{i=1}^{n} \text{Bernoulli}(y_i|\mu_i) N(\beta|0, \tau W)\, d\beta$$

Unlike above we cannot compute the integral in closed form. Instead we use an argument similar to [6]. To make this problem tractable we need to take two Taylor approximations. First approximate $p(y|\beta) = \exp\{\log p(y|\beta)\}$ using a Taylor expansion around $\beta = 0$, the hypothesis we want to test. Taking gradients with respect to β

$$\begin{aligned}
\log p(y|\beta) &= \sum_{i=1}^{n} y_i \log \mu_i + (1-y_i)\log(1-\mu_i) \\
\implies \nabla_\beta \log p(y|\beta) &= \sum_{i=1}^{n} y_i \frac{1}{\mu_i}\mu_i(1-\mu_i)g_i - (1-y_i)\frac{1}{1-\mu_i}\mu_i(1-\mu_i)g_i \\
&= \sum_{i=1}^{n} (y_i - \mu_i) g_i \\
&= G^T(y-\mu) \\
\implies \nabla\nabla_\beta \log p(y|\beta) &= G^T V G \\
V &:= \text{diag}(\mu_1(1-\mu_1), ..., \mu_n(1-\mu_n))
\end{aligned}$$

Then we can compute

$$\begin{aligned}
\nabla_\beta \exp\{\log p(y|\beta)\} &= \exp\{\log p(y|\beta)\} \nabla_\beta \log p(y|\beta) \\
\nabla\nabla_\beta \exp\{\log p(y|\beta)\} &= \exp\{\log p(y|\beta)\} \left(\nabla_\beta \log p(y|\beta) \nabla_\beta \log p(y|\beta)^T + \nabla\nabla_\beta \log p(y|\beta)\right)
\end{aligned}$$

Expanding around $\beta = 0$

$$\exp\{\log p(y|\beta)\} \approx \exp\{\log p(y|\beta=0)\} \left(1 + (y-\mu)^T G\beta + \frac{1}{2}\beta^T \left(G^T(y-\mu)(y-\mu)^T G + G^T V G\right)\beta\right)$$

where $\text{logit}(\mu) = X\beta$. Then we can write the integral as an expectation

$$L(\alpha, \tau) = E_\beta\left[\exp\{\log p(y|\beta)\}\right] \approx p(y|\beta=0)\left(1 + \frac{1}{2}\text{Tr}(\tau G^T(y-\mu)(y-\mu)^T G W + \tau G^T V G W)\right)$$

Taking another Taylor expansion around $\tau = 0$ of the log likelihood

$$\begin{aligned}
l(\alpha, \tau) := \log p(y) &= \log p(y|\beta=0) + \log\left(1 + \frac{\tau}{2}\text{Tr}(G^T(y-\mu)(y-\mu)^T G W + G^T V G W)\right) \\
&\approx \log p(y|\beta=0) + \frac{\tau}{2}\text{Tr}\left(G^T(y-\mu)(y-\mu)^T G W + G^T V G W\right)
\end{aligned}$$

Rearranging terms using the "trace trick"

$$\begin{aligned}
l(\alpha, \tau) &\approx \log p(y|\beta=0) + \frac{\tau}{2}\text{Tr}(G^T(y-\mu)(y-\mu)^T G W) + \frac{\tau}{2}\text{Tr}(G^T V G W) \\
&= \log p(y|\beta=0) + \frac{\tau}{2}(y-\mu)^T G W G^T (y-\mu) + \frac{\tau}{2}\text{Tr}(G^T V G W)
\end{aligned}$$

Now we can compute the score statistic as before

$$\frac{\partial l(\alpha, \tau)}{\partial \tau} = \frac{\partial}{\partial \tau}\log p(y) = \frac{1}{2}(y-\mu)^T G W G^T (y-\mu) + \frac{1}{2}\text{Tr}(G^T V G W)$$

Again as before, the first term is the only random term. Rescaling by $\frac{1}{2}$ gives us the result

$$Q = (y-\mu)^T G W G^T (y-\mu)$$

1.3 Aggregated Cauchy association test (ACATV)

The aggregated Cauchy association test (ACAT) is a method for combining p-values across multiple tests [7]. Similar to Fisher's method which transforms p-values to χ^2 random variables, or Stouffer's method which transforms p-values to Z scores, ACAT transforms p-values to Cauchy distributed random variables. It uses the fact that under some mild assumptions, convex combinations of Cauchy transformed p-values will follow a standard Cauchy distribution — even if the p-values are correlated. If $p_1, ..., p_d$ are the p-values to be combine and $w_1, ..., w_d$ the weights, then the test statistic is

$$T = \sum_{i=1}^{d} w_i \tan\{(0.5 - p_i)\pi\} \sim \text{Cauchy}(0, 1)$$

When the weights are all equal ACAT can be used to combine p-values from correlated tests to correct for multiple testing. In the context of rare variant association testing, p-values can be combined into a set-based test by grouping variables in an annotation category [8]. Like the WST and SKATO, per variant weights can be chosen based on features of the variant like its minor allele frequency. If w_j is the weight for a variant j in WST or SKATO, then for ACATV others have suggested [8] to set weight to be

$$\begin{aligned} w_{j,ACATV} &= w_j^2 f_j (1 - f_j) \bar{w} \\ \bar{w} &= \sum_j w_{j,ACATV} \end{aligned}$$

Thus

$$T_{ACATV} = \frac{1}{\bar{w}} \sum_{j=1}^{p} w_{j,ACATV} \tan\{(0.5 - p_j)\pi\}$$

1.4 Saddlepoint approximation for unbalanced binary traits

For unbalanced binary using a normal approximation to compute the distribution of the test statistic can lead to inflated type I error. In this setting, saddlepoint approximation (SPA) [9] has been shown to help control type I error [10]. SPA uses the cumulant-generating function of the test statistic to approximate its distribution. The test statistic for a score test in logistic regression is

$$S_j = \sum_{i=1}^{n} g_{ij}(y_i - \hat{\mu_i})$$

The cumulant generating function is

$$\begin{aligned} K(t) &= \log\left(\mathbb{E}_{H_0}[e^{tS_j}]\right) \\ &= \sum_{i=1}^{n} \log(1 - \hat{\mu_i} + \hat{\mu_i} e^{g_{ij}t}) - t \sum_{i=1}^{n} g_{ij}\hat{\mu_i} \end{aligned}$$

where $\hat{\mu_i}$ is estimated under the null hypothesis. The first two derivatives of $K(t)$ are

$$\begin{aligned} K'(t) &= \sum_{i=1}^{n} \frac{\hat{\mu_i} g_{ij}}{(1 - \hat{\mu_i})e^{-g_{ij}t} + \hat{\mu_i}} - \sum_{i=1}^{n} g_{ij}\hat{\mu_i} \\ K''(t) &= \frac{\hat{\mu_i}(1 - \hat{\mu_i}) g_{ij}^2 e^{-g_{ij}t}}{\left((1 - \hat{\mu_i})e^{-g_{ij}t} + \hat{\mu_i}\right)^2} \end{aligned}$$

Then the saddlepoint approximation to the distribution of S_j is given by

$$\begin{aligned} \Pr(S_j < s) &= \Phi\left(w + \frac{1}{w} \log\left(\frac{v}{w}\right)\right) \\ w &= \text{sign}(\delta^*)\sqrt{2(\delta^* s - K(\delta^*)} \\ v &= \delta^* \sqrt{K''(\delta^*)} \end{aligned}$$

where Φ is the standard normal distribution and δ^* is the solution to $K'(t) = s$.

When only an intercept is included in the model, a SPA can be computed from case-control counts and genotype counts alone. The intercept is given by $\hat{\mu_i} = \hat{\mu} = \frac{\text{\# cases}}{\text{\# cases+\# controls}}$. If we let n_{ref}, n_{het} and n_{alt} be the number of individuals with homozygous reference, heterozygous, and homozygous alternate genotypes respectively, we have

$$K(t) = \sum_{x \in \{ref, het, alt\}} n_x \log(1 - \hat{\mu} + \hat{\mu} e^{g_x t}) \tag{1}$$

where g_x are the (possibly centered and standardized) reference, heterozygous, and homozygous alternate genotypes. This approach has been shown to work well in the setting of single-variant meta-analysis [11].

To extend to gene-based tests, we use the observation the sum test approximates the collapsing burden test, and that we can compute genotype counts from the collapsing burden test. Let n^s_{cases} and $n^s_{controls}$ be the number of cases and controls for a study s. Furthermore, let g^s_x be the number of genotype counts for $x \in$ {hom ref, het, hom alt}. We compute

$$\hat{\mu} = \frac{\sum_s n^s_{cases}}{\sum_s n^s_{cases} + n^s_{controls}}$$
$$n_x = \sum_s n^s_x \text{ for } x \in \{\text{hom ref, het, hom alt}\}$$

We then use cumulant generating function in equation 1 to compute a p-value for the sum test.

For the WST and SKATO, we use the burden p-value computed by SPA to compute a calibration factor similar to [12]. Specifically, let p_{ST} and p_{SPA} be the p-values computed from the sum test and SPA respectively. Then

$$r = \max\left(1, \frac{\chi^2_{quantile}(1 - p_{ST})}{\chi^2_{quantile}(1 - p_{SPA})}\right)$$

We use r as a calibration factor to increase the covariance of the score statistics by replacing $\text{Cov}(S)$ with $r\text{Cov}(S)$.

For single variants, we can use the p-value computed by SPA to compute a per variant calibration factor [13]. Let p_{NM} and p_{SPA} be the p-values computed from a normal approximation and SPA respectively for a single variant. Then

$$r_j = \max\left(1, \frac{\chi^2_{quantile}(1 - p_{NM})}{\chi^2_{quantile}(1 - p_{SPA})}\right)$$

We use r_j as a calibration factor to increase the variance of the score statistics by replacing $\text{Cov}(S_j)$ with $r_j\text{Cov}(S_j)$.

1.5 Estimating mask genotype counts and allele frequencies

1.5.1 Preliminaries

We start with a result that will be useful for estimating genotype counts of burden masks. Let $G_1 = X_1^{(1)} + X_1^{(2)}$ and $G_2 = X_2^{(1)} + X_2^{(2)}$ be two genotypes made up of the variants $X_j^{(h)}$ on haplotypes $h \in \{1, 2\}$. We want to compute $\Pr(X_1^{(h)} = 1, X_2^{(h)} = 1)$ from the LD between G_1 and G_2.

Assuming each haplotype is inherited independently, then $\mathbb{E}\left[X_1^{(h)} X_2^{(k)}\right] = \mathbb{E}\left[X_1^{(h)}\right] \mathbb{E}\left[X_2^{(k)}\right]$ for $h \neq k$.

Let f_1 and f_2 be the frequencies of variants 1 and 2. We have

$$\begin{aligned}
\mathrm{Cov}(G_1, G_2) &= \mathrm{Cov}\left(X_1^{(1)} + X_1^{(2)}, X_2^{(1)} + X_2^{(2)}\right) \\
&= \mathbb{E}\left[\left(X_1^{(1)} + X_1^{(2)}\right)\left(X_2^{(1)} + X_2^{(2)}\right)\right] - \mathbb{E}\left[X_1^{(1)} + X_1^{(2)}\right]\mathbb{E}\left[X_2^{(1)} + X_2^{(2)}\right] \\
&= \mathbb{E}\left[X_1^{(1)}X_2^{(1)}\right] + \mathbb{E}\left[X_1^{(1)}X_2^{(2)}\right] + \mathbb{E}\left[X_1^{(2)}X_2^{(1)}\right] + \mathbb{E}\left[X_1^{(2)}X_2^{(2)}\right] - 4f_1f_2 \\
&= \mathbb{E}\left[X_1^{(1)}X_2^{(1)}\right] + \mathbb{E}\left[X_1^{(1)}\right]\mathbb{E}\left[X_2^{(2)}\right] + \mathbb{E}\left[X_1^{(2)}\right]\mathbb{E}\left[X_2^{(1)}\right] + \mathbb{E}\left[X_1^{(2)}X_2^{(2)}\right] - 4f_1f_2 \\
&= \mathbb{E}\left[X_1^{(1)}X_2^{(1)}\right] + \mathbb{E}\left[X_1^{(2)}\right]\mathbb{E}\left[X_2^{(1)}\right] - 2f_1f_2 \\
&= 2\mathbb{E}\left[X_1^{(1)}X_2^{(1)}\right] - 2f_1f_2 \\
&= 2\mathbb{E}\left[X_1X_2\right] - 2f_1f_2 \\
\implies \Pr(X_1 = 1, X_2 = 1) = \mathbb{E}[X_1X_2] &= \frac{1}{2}\mathrm{Cov}(G_1, G_2) + f_1f_2
\end{aligned}$$

1.5.2 Genotype counts

We derived an estimator for the genotype counts of a burden mask from the genotype counts of the variants in the mask and their LD. Let G_{ji} be the genotype of variant j in individual i. Let $N_{G_j=1}$ and $N_{G_j=2}$ be the heterozygous and homozygous genotype counts respectively. We want to find some combination of the $N_{G_j=1}$ and $N_{G_j=2}$ to estimate the heterozygote and homozygote genotype counts of the mask. Focusing on heterozygotes (the homozygous case is similar), if $Y_i = \max\{G_{1i}, ..., G_{pi}\}$ is the mask genotype we want to find coefficients c_m that

$$N_{Y_i=1} = \sum_{j=1}^{p} c_j N_{G_j=1}$$

One possible strategy is to compute the c_j sequentially. If we set $c_1 = 1$, then for each c_{j+1} we can compute

$$\begin{aligned}
\Pr(G_1 \neq 1, G_2 \neq 1, ..., G_j \neq 1, G_{j+1} = 1) &= \Pr(G_1 \neq 1, G_2 \neq 1, ..., G_j \neq 1 | G_{j+1} = 1)\Pr(G_{j+1} = 1) \\
&= \Pr(G_1 \neq 1, G_2 \neq 1, ..., G_j \neq 1 | G_{j+1} = 1)\frac{N_{G_j=1}}{N}
\end{aligned}$$

This gives us the proportion of heterozygotes at G_{j+1} that we have not already counted among $G_1, ..., G_j$. The coefficient we want it

$$c_{j+1} = \Pr(G_1 \neq 1, G_2 \neq 1, ..., G_j \neq 1 | G_{j+1} = 1)$$

If we assume that all LD among $G_1, ..., G_j$ is explained by G_{j+1}, then we can approximate

$$\Pr(G_1 \neq 1, G_2 \neq 1, ..., G_j \neq 1 | G_{j+1} = 1) \approx \prod_{m=1}^{j} \Pr(G_m \neq 1 | G_{j+1} = 1)$$

Let f_j be the allele frequency of variant j, and suppose that $X_j^{(1)} = 1$ and $X_j^{(2)} = 0$ are the haplotypes for variant j (the labeling of the haplotype is arbitrary). We have

$$\Pr(G_m = 1 | G_j = 1) = \Pr(X_m^{(1)} = 1 | X_j^{(1)} = 1)(1 - f_j) + (1 - \Pr(X_m^{(1)} = 1 | X_j^{(1)} = 1))f_j$$

The first term is the case where $G_j = 1$ and $G_m = 1$ along the same haplotype, the second term is when $G_j = 1$ and $G_m = 1$ along different haplotypes. This gives us

$$\Pr(G_m \neq 1 | G_j = 1) = 1 - \Pr(G_m = 1 | G_j = 1)$$

The argument for homozygotes is similar. We want to compute

$$\begin{aligned}\Pr(G_m = 2|G_j = 2) &= \Pr(X_m^{(1)} = 1|X_j^{(1)} = 1)\Pr(X_m^{(2)} = 1|X_j^{(2)} = 1) \\ &= \Pr(X_m^{(1)} = 1|X_j^{(1)} = 1)^2\end{aligned}$$

So

$$\Pr(G_j \neq 2|G_m = 2) = 1 - \Pr(G_j = 2|G_m = 2)$$

1.5.3 Estimating allele frequencies when genotype counts are unavailable

When genotype counts are unavailable, we can estimate the allele frequency of burden masks from the allele frequencies of variants in the mask and their LD. To derive the estimator we model the haplotype of the variants contributing to the mask. Suppose $X_1, ..., X_p$ where $X_j \in \{0, 1\}$ are the variants in the haplotype constributing to the mask. The mask haplotype is $Y = \max\{X_1, ..., X_p\}$. The frequency of the haplotype is

$$\begin{aligned}\mathbb{E}[Y] &= \Pr(Y = 1) = 1 - \Pr(Y = 0) \\ &= 1 - \Pr(X_1 = 0, ..., X_p = 0)\end{aligned}$$

If the mask is in Hardy-Weinberg equilibrium, then the frequency of the mask haplotype is equivalent to the frequency of the mask. We want to approximate $\Pr(X_1 = 0, ..., X_p = 0)$ from something computable from the LD matrix. If we model the variants in the mask as a Markov chain, then this term becomes

$$\Pr(X_1 = 0, ..., X_p = 0) \approx \Pr(X_1 = 0) \prod_{t=2}^{T} \Pr(X_t = 0|X_{t-1} = 0)$$

The motivation for this choice is that $\Pr(X_t = 0|X_{t-1} = 0)$ can be computed from the LD matrix. Specifically

$$\begin{aligned}\Pr(X_t = 0|X_{t-1} = 0) &= \frac{\Pr(X_t = 0, X_{t-1} = 0)}{f_{t-1}} \\ &= \frac{1}{f_{t-1}} (1 - \Pr(X_t = 1) - \Pr(X_{t-1} = 1) + \Pr(X_t = 1, X_{t-1} = 1))\end{aligned}$$

What remains is to pick an order to evaluate the Markov chain. We start by setting X_1 to the most frequent variant. For each subsequent variant we choose the variant in the most LD with the previous. Thus for X_2, we choose the variant with the most LD to X_1.

References

[1] Bingshan Li and Suzanne M Leal. "Methods for detecting associations with rare variants for common diseases: application to analysis of sequence data". In: *The American Journal of Human Genetics* 83.3 (2008), pp. 311–321.

[2] Bo Eskerod Madsen and Sharon R Browning. "A groupwise association test for rare mutations using a weighted sum statistic". In: *PLoS genetics* 5.2 (2009), e1000384.

[3] Seunggeung Lee et al. "Rare-variant association analysis: study designs and statistical tests". In: *The American Journal of Human Genetics* 95.1 (2014), pp. 5–23.

[4] Dan-Yu Lin and Patrick F Sullivan. "Meta-analysis of genome-wide association studies with overlapping subjects". In: *The American Journal of Human Genetics* 85.6 (2009), pp. 862–872.

[5] Christopher M Bishop. *Pattern recognition and machine learning*. Springer, 2006.

[6] Xihong Lin. "Variance component testing in generalised linear models with random effects". In: *Biometrika* 84.2 (1997), pp. 309–326.

[7] Yaowu Liu and Jun Xie. "Cauchy combination test: a powerful test with analytic p-value calculation under arbitrary dependency structures". In: *Journal of the American Statistical Association* (2019).

[8] Yaowu Liu et al. "ACAT: a fast and powerful p value combination method for rare-variant analysis in sequencing studies". In: *The American Journal of Human Genetics* 104.3 (2019), pp. 410–421.

[9] Rounak Dey et al. "A fast and accurate algorithm to test for binary phenotypes and its application to PheWAS". In: *The American Journal of Human Genetics* 101.1 (2017), pp. 37–49.

[10] Wei Zhou et al. "Scalable generalized linear mixed model for region-based association tests in large biobanks and cohorts". In: *Nature genetics* 52.6 (2020), pp. 634–639.

[11] Rounak Dey et al. "Robust meta-analysis of biobank-based genome-wide association studies with unbalanced binary phenotypes". In: *Genetic epidemiology* 43.5 (2019), pp. 462–476.

[12] Zhangchen Zhao et al. "UK Biobank whole-exome sequence binary phenome analysis with robust region-based rare-variant test". In: *The American Journal of Human Genetics* 106.1 (2020), pp. 3–12.

[13] Eunjae Park et al. "Meta-SAIGE: Scalable and Accurate Meta-Analysis for Rare Variants". In: *medRxiv* (2024), pp. 2024–09.

2 Supplementary Figures

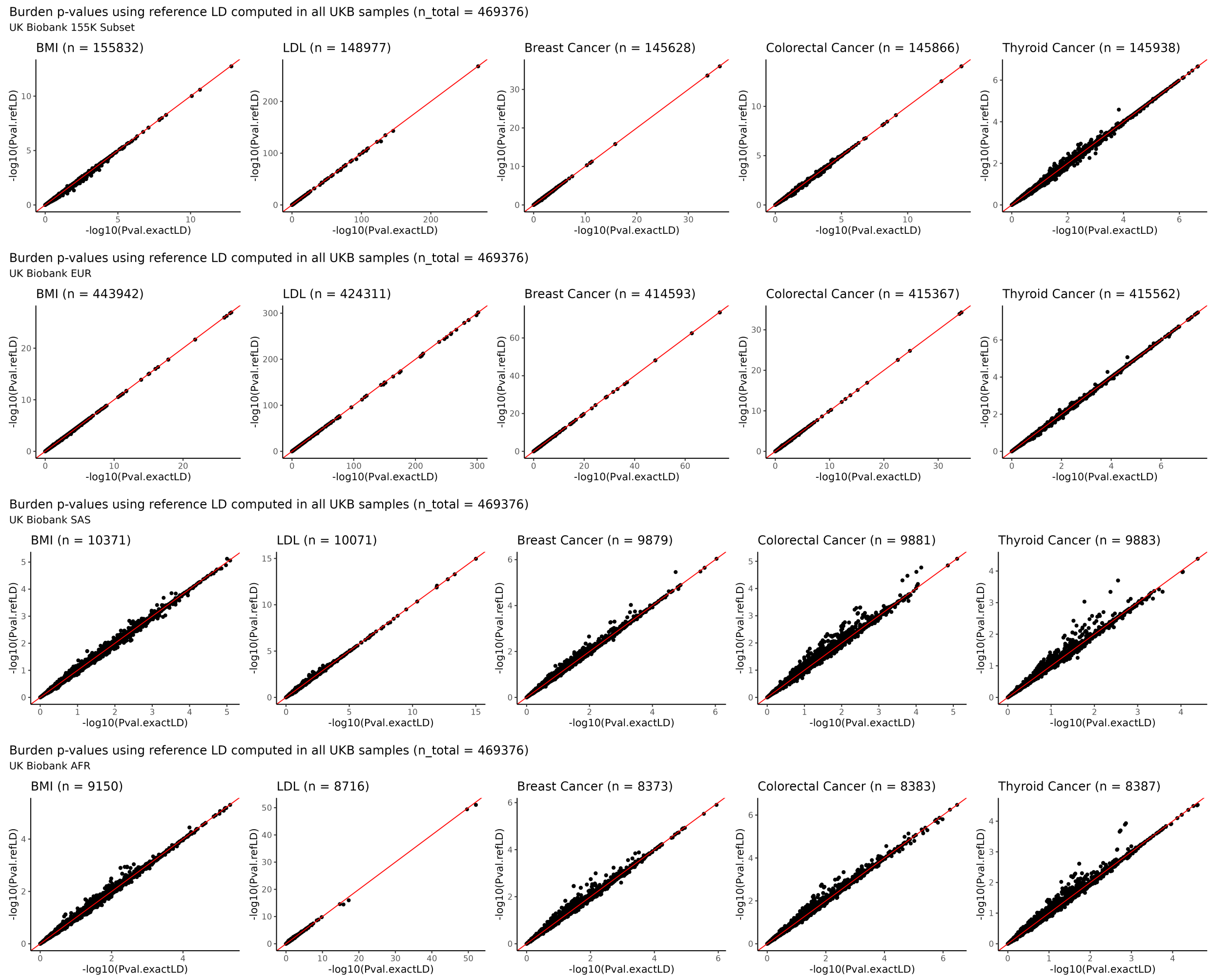

Supplementary Figure 1: Scatterplots comparing p-values of burden tests computed using the exact covariance of the score statistics and a reference LD matrix computed in UK Biobank ALL. The panels compare p-values computed using the covariance of the score statistics in the sample (x-axis; Pval.exactLD; sample sizes given above each panel) to p-values computed using a reference LD panel computed in all samples in UK Biobank (y-axis; Pval.refLD; sample sizes given by n_total).

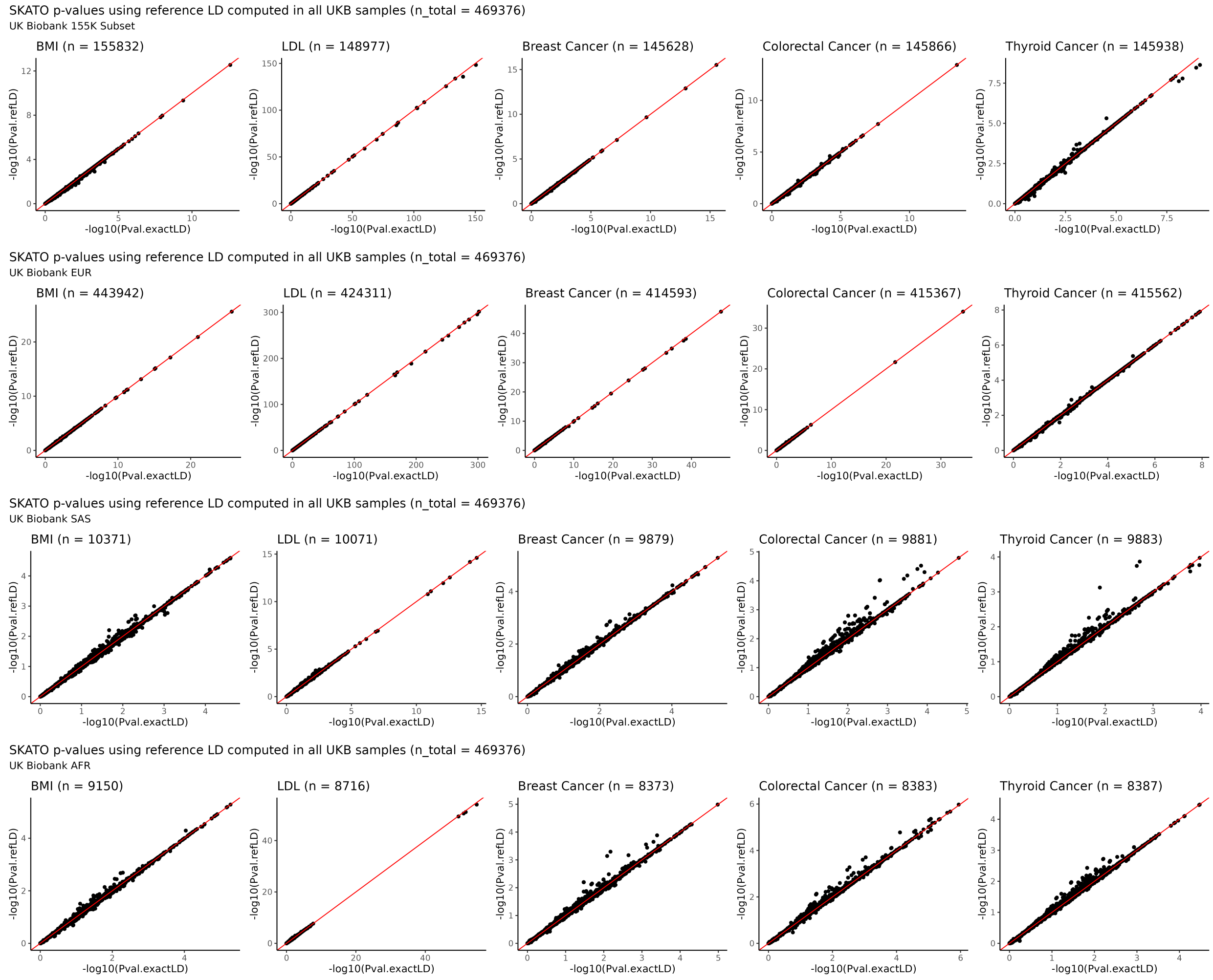

Supplementary Figure 2: **Scatterplots comparing SKATO *p*-values computed using the exact covariance of the score statistics and a reference LD matrix computed in UK Biobank ALL.** The panels compare *p*-values computed using the covariance of the score statistics in the sample (x-axis; Pval.exactLD; sample sizes given above each panel) to *p*-values computed using a reference LD panel computed in all samples in UK Biobank (y-axis; Pval.refLD; sample sizes given by n_total).

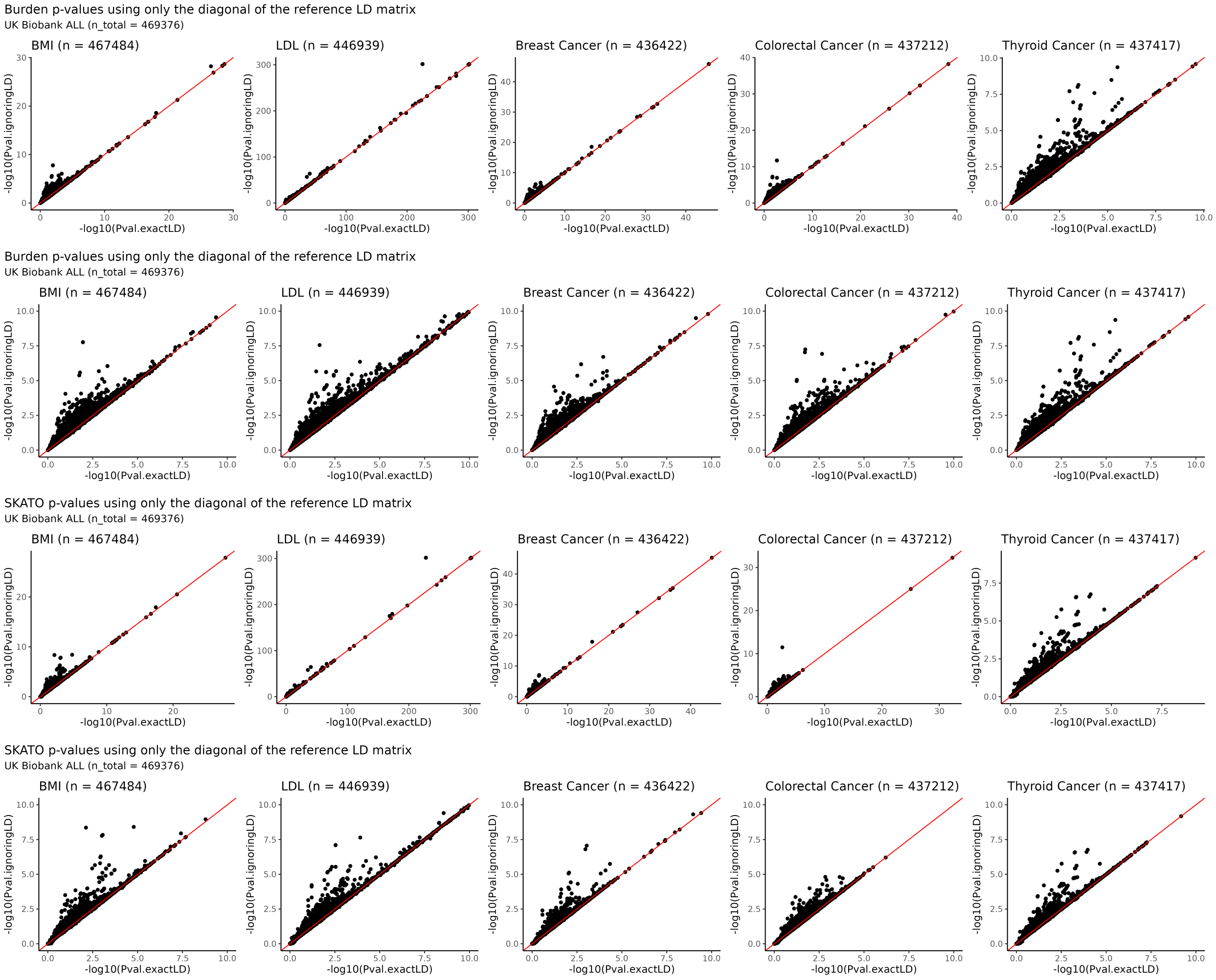

Supplementary Figure 3: Scatterplots comparing burden test and SKATO p-values computed using the exact covariance of the score statistics p-values computed by ignoring LD between variants. The top two rows display p-values for burden tests (note the change in scale between rows 1 and 2), and the bottom two rows display p-values for SKATO. The panels compare p-values computed using the covariance of the score statistics in the sample (x-axis; Pval.exactLD; sample sizes given above each panel) to p-values computed ignoring LD (y-axis; Pval.ignoreLD).

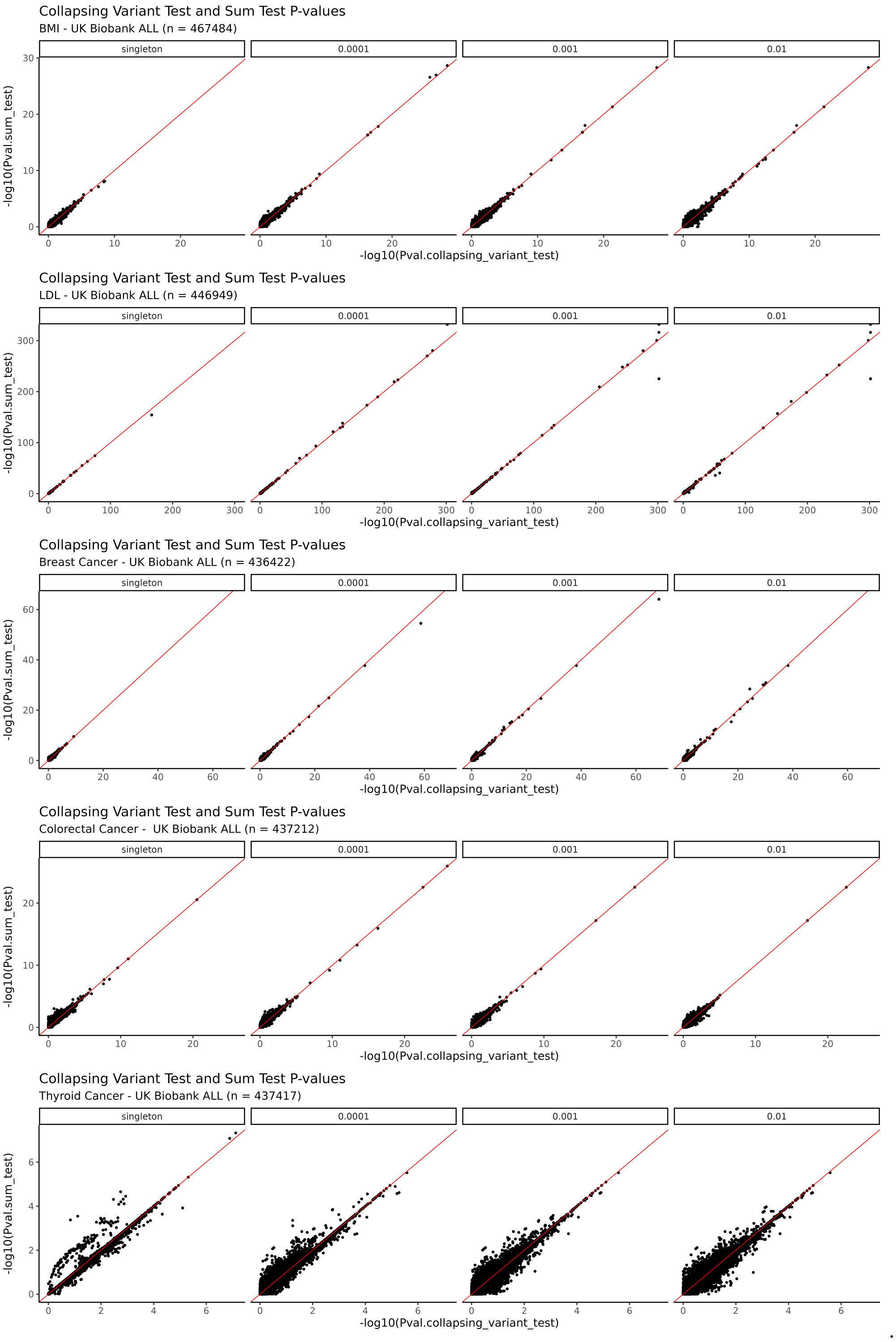

Supplementary Figure 4: **Scatterplot comparing p-values from the collapsing variant test and the sum test across 5 traits in UK Biobank ALL.** Each row corresponds to a trait, and each column corresponds to an AAF bin for a burden test. Each panel includes burden masks computed across 7 annotation categories. Burden testing for the collapsing variant test and sum test was performed in REGENIE using the –build-mask max and –build-mask sum options respectively.

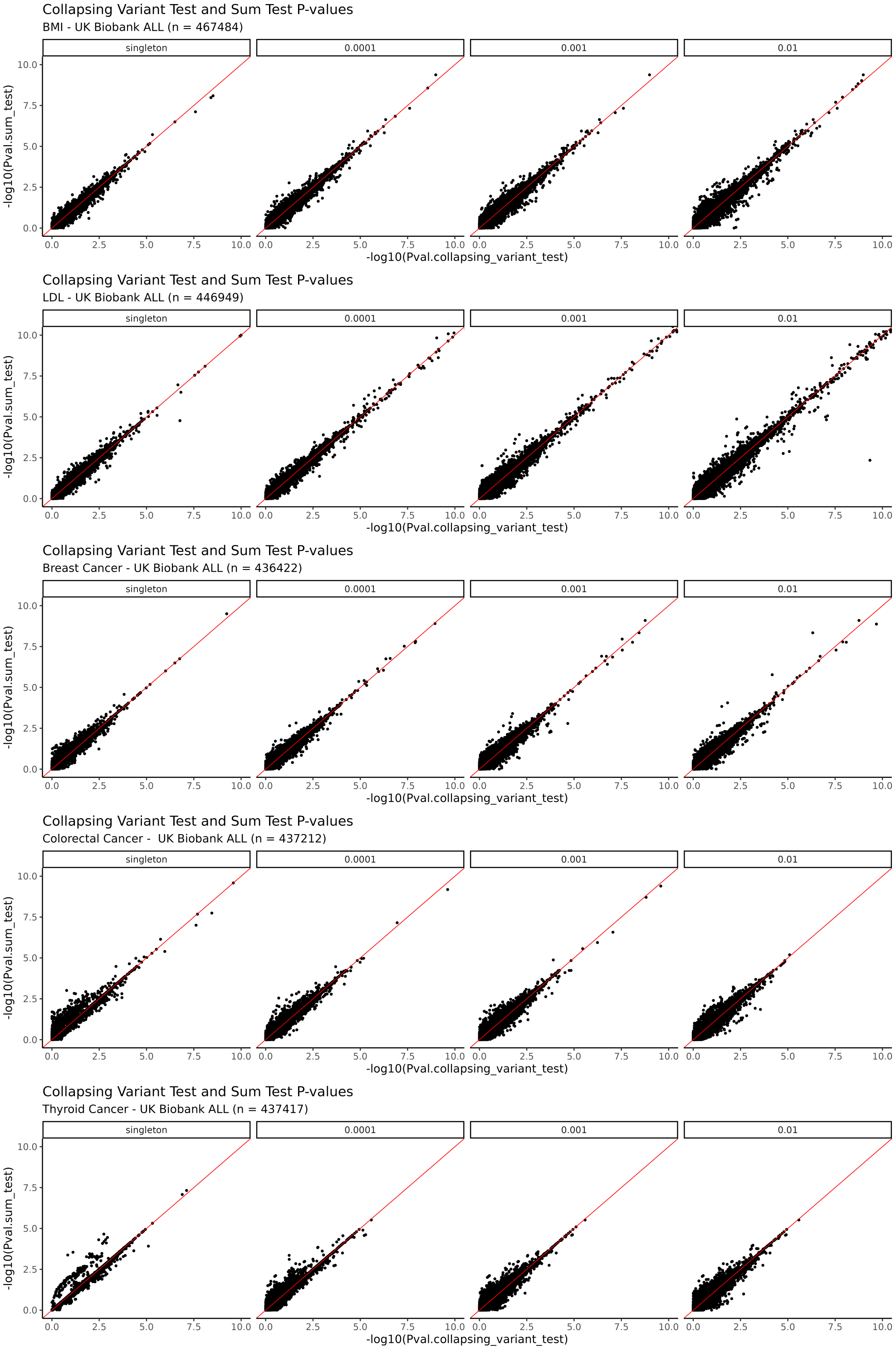

Supplementary Figure 5: Zoomed in scatterplot comparing p-values from the collapsing variant test and the sum test. Each row corresponds to a trait, and each column corresponds to an AAF bin for a burden test. Each panel includes burden masks computed across 7 annotation categories for each gene. Burden testing for the collapsing variant test and sum test was performed in REGENIE using the –build-mask max and –build-mask sum options respectively.

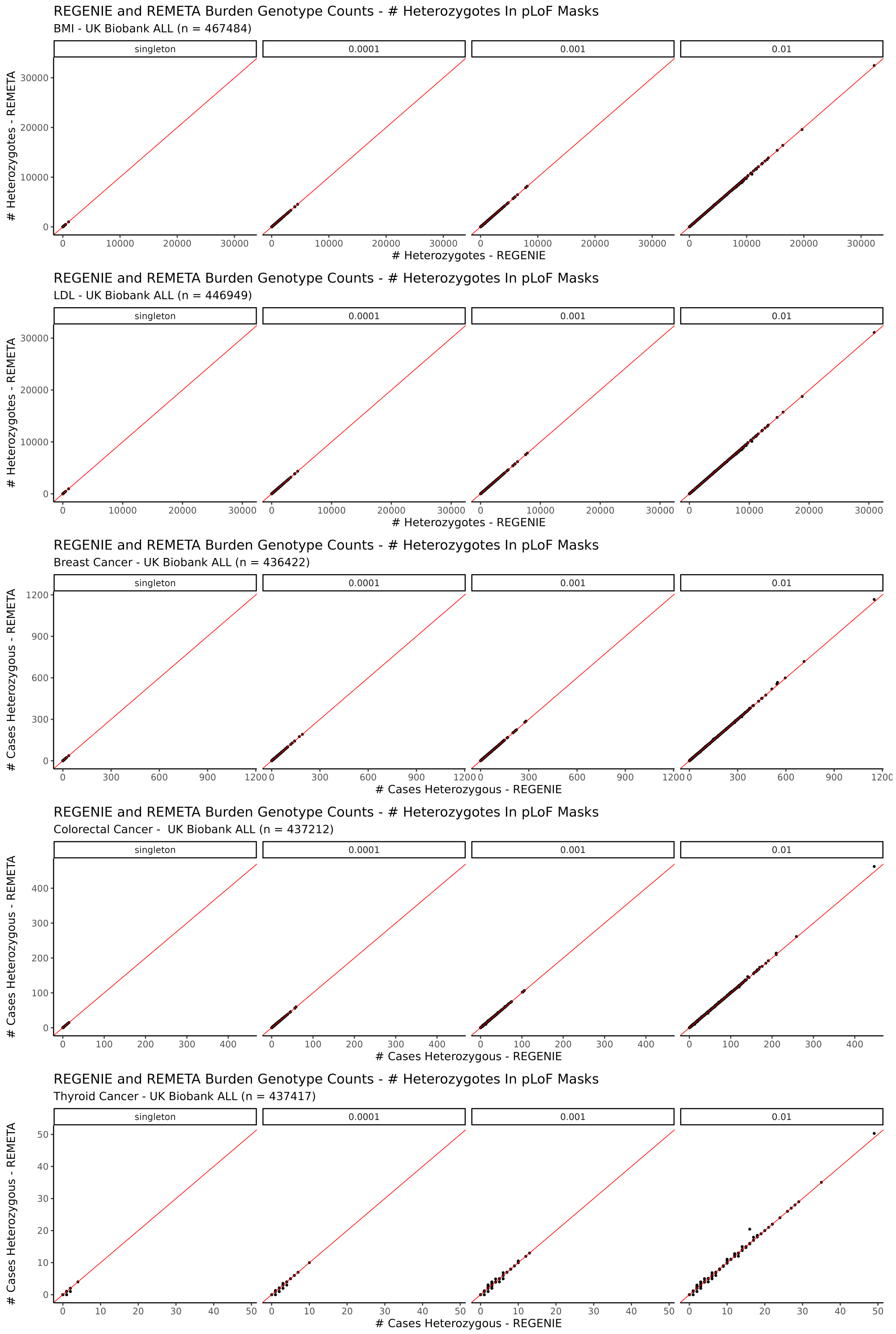

Supplementary Figure 6: **Scatterplot comparing genotype counts of burden masks computed by REGENIE and estimated by REMETA in pLoF masks.** Each row corresponds to a trait, and each column corresponds to an AAF bin for a burden test. Each panel includes only the pLoF mask for each gene. For quantitative traits, genotypes counts in the whole sample are displayed. For binary traits, genotype counts among cases are displayed.

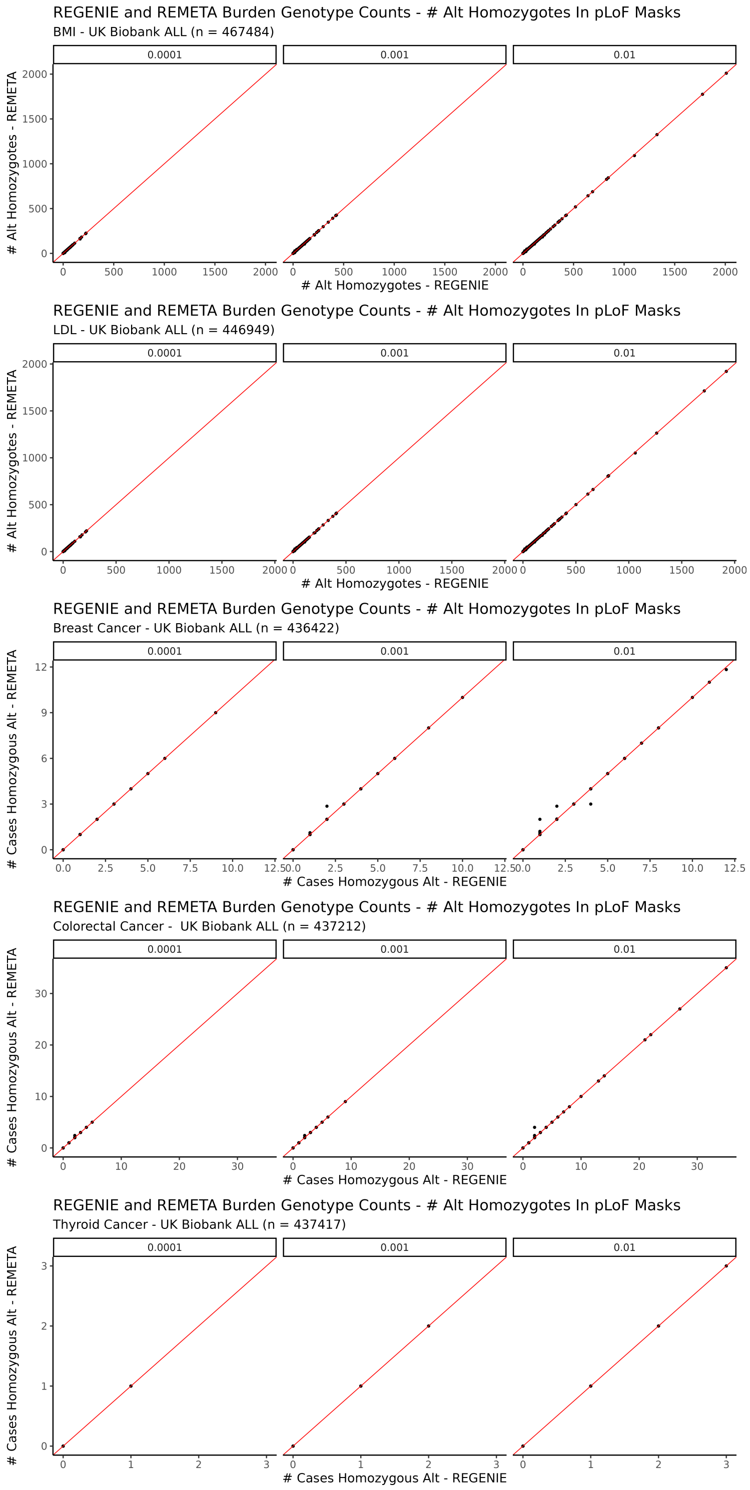

Supplementary Figure 7: **Scatterplot comparing genotype counts of burden masks computed by REGENIE and estimated by REMETA.** Each row corresponds to a trait, and each column corresponds to an AAF bin for a burden test. For quantitative traits, genotypes counts in the whole sample are displayed. For binary traits, genotype counts among cases are displayed. Each panel includes only the pLoF mask for each gene.

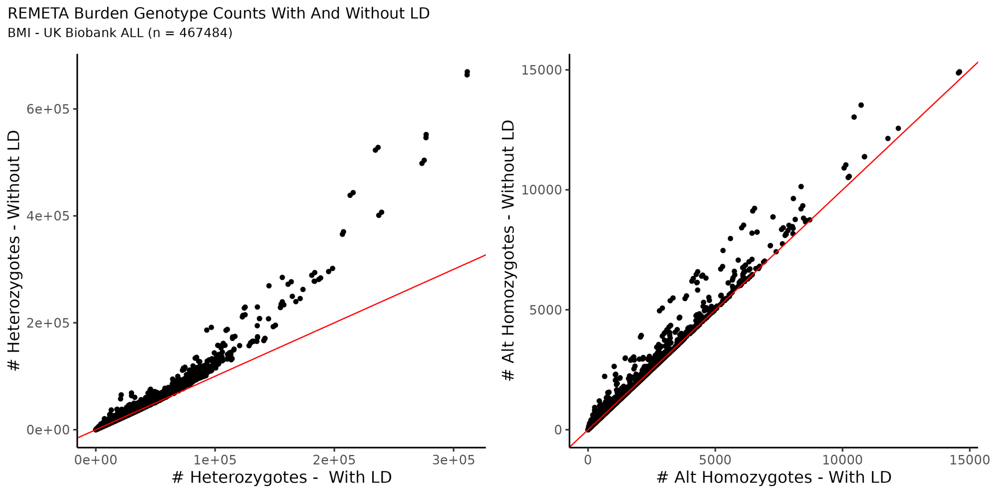

Supplementary Figure 8: **Scatterplot comparing genotype counts of burden masks estimated with REMETA to a naive estimate that ignores LD in BMI.** Estimates that ignore LD are equivalent to summing the genotype counts of the variants in mask.

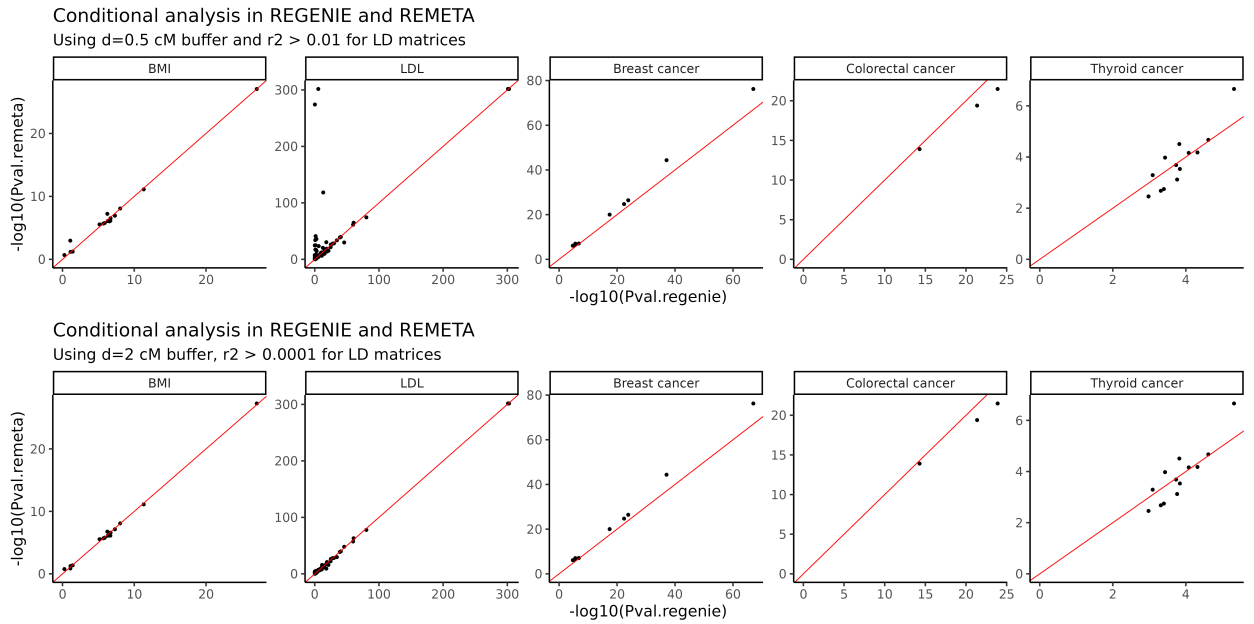

Supplementary Figure 9: Scatterplot of GENE P p-values from conditional analysis computed across two sets of parameters for LD matrix generation. Top: Conditional analysis in REMETA is performed using LD matrices with a 0.5 cM buffer storing entries with $r^2 > 0.01$. Bottom: Conditional analysis in REMETA is performed using LD matrices with a 2 cM buffer storing entries with $r^2 > 0.0001$. Conditional analysis was performed for all 157 ExWAS significant gene-trait pairs in the marginal analysis. Except for LDL, most p-values computed by REMETA are similar between both parameter sets.

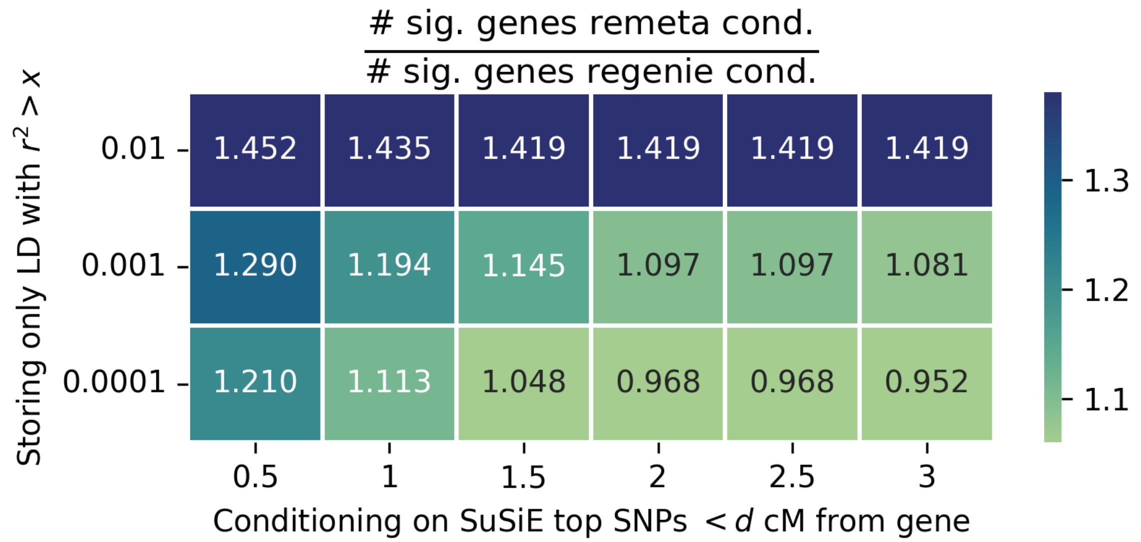

Supplementary Figure 10: **Comparison of conditional analysis in REMETA to REGENIE when conditioning on top SNPs from variants fine-mapped with SuSiE.** The heatmap displays the ratio of number of ExWAS significant GENE P p-values in REMETA to REGENIE when conditioning on variants with the top cPIP in each credible set. Association testing was performed for all 157 ExWAS significant gene-trait pairs in the marginal analysis.

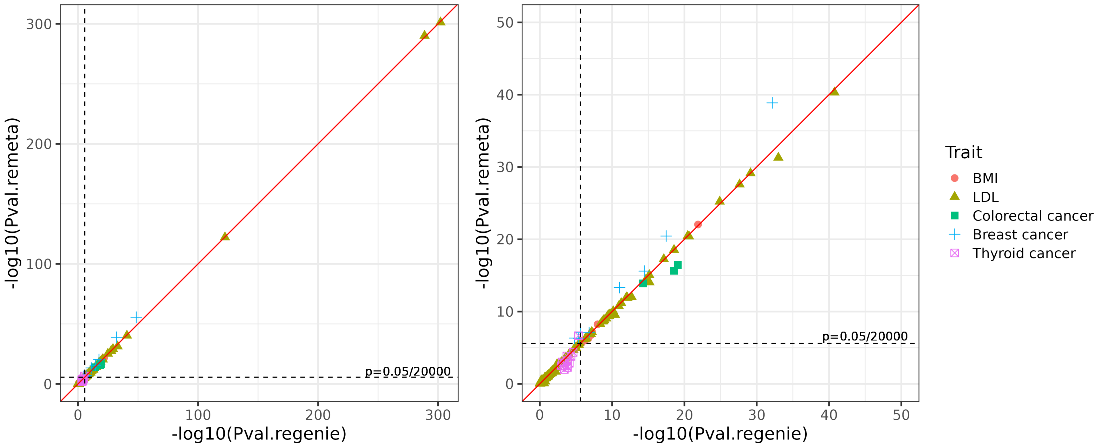

Supplementary Figure 11: **Evaluation of an alternate strategy for conditional analysis in REMETA.** The x-axis displays GENE P p-values for gene-based tests when running REGENIE conditional on variants with the top cPIP per credible set after fine-mapping with SuSiE. The y-axis displays GENE P p-values for gene-based tests when running REMETA using summary statistics from conditional analysis in REGENIE and LD matrices for marginal association testing. Gene-based tests were performed on 157 gene-trait pairs that were ExWAS significant in the marginal analysis.

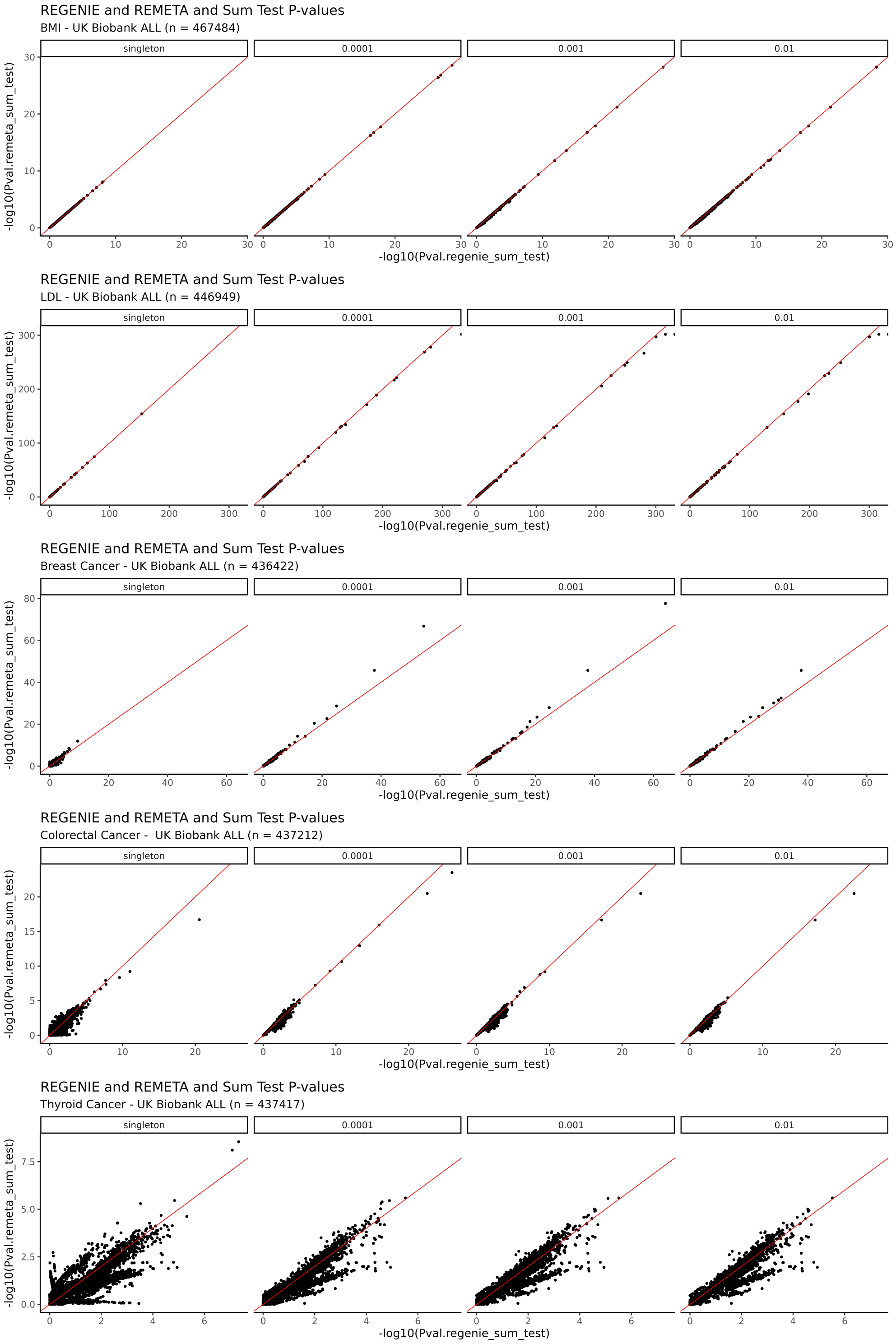

Supplementary Figure 12: **Scatterplot comparing** p**-values from the sum test computed using REGENIE and the sum test computed using REMETA.** Each row corresponds to a trait, and each column corresponds to an AAF bin for a burden test. Each panel includes burden masks computed across 7 annotation categories for each gene. Burden testing for the collapsing variant test and sum test was performed in REGENIE using the –build-mask sum option.

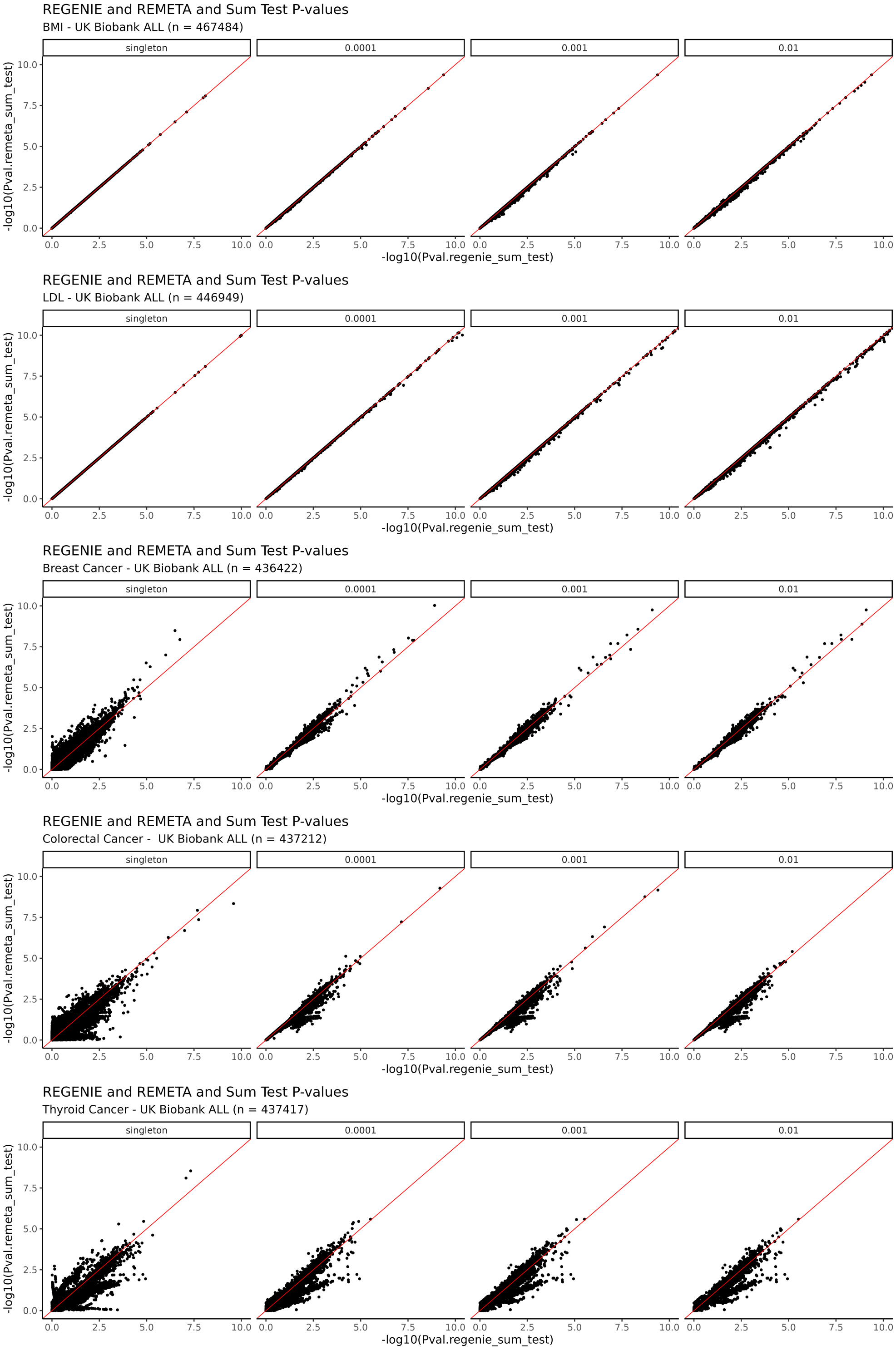

Supplementary Figure 13: **Zoomed-in scatterplot comparing p-values from the sum test computed using REGENIE and the sum test computed using REMETA.** Each row corresponds to a trait, and each column corresponds to an AAF bin for a burden test. Each panel includes burden tests computed across 7 annotation categories for each gene. Burden testing for the collapsing variant test and sum test was performed in REGENIE using the –build-mask sum option.

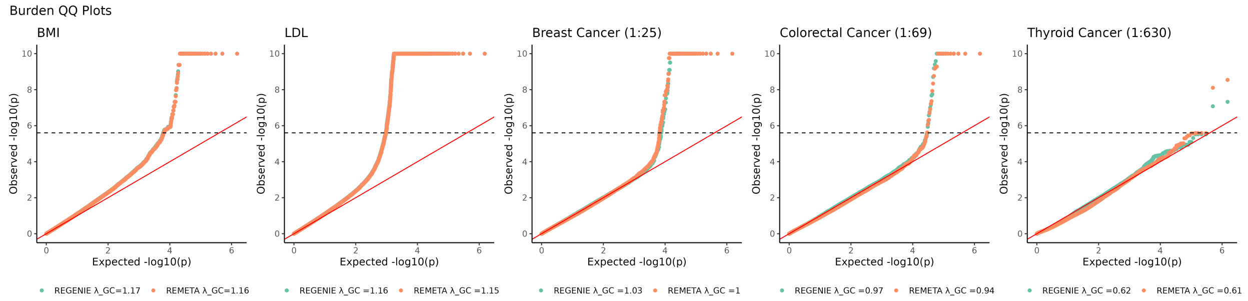

Supplementary Figure 14: **QQ plots from the sum test in REGENIE and REMETA across 5 traits in UK Biobank ALL.** λ_{GC}: genomic control.

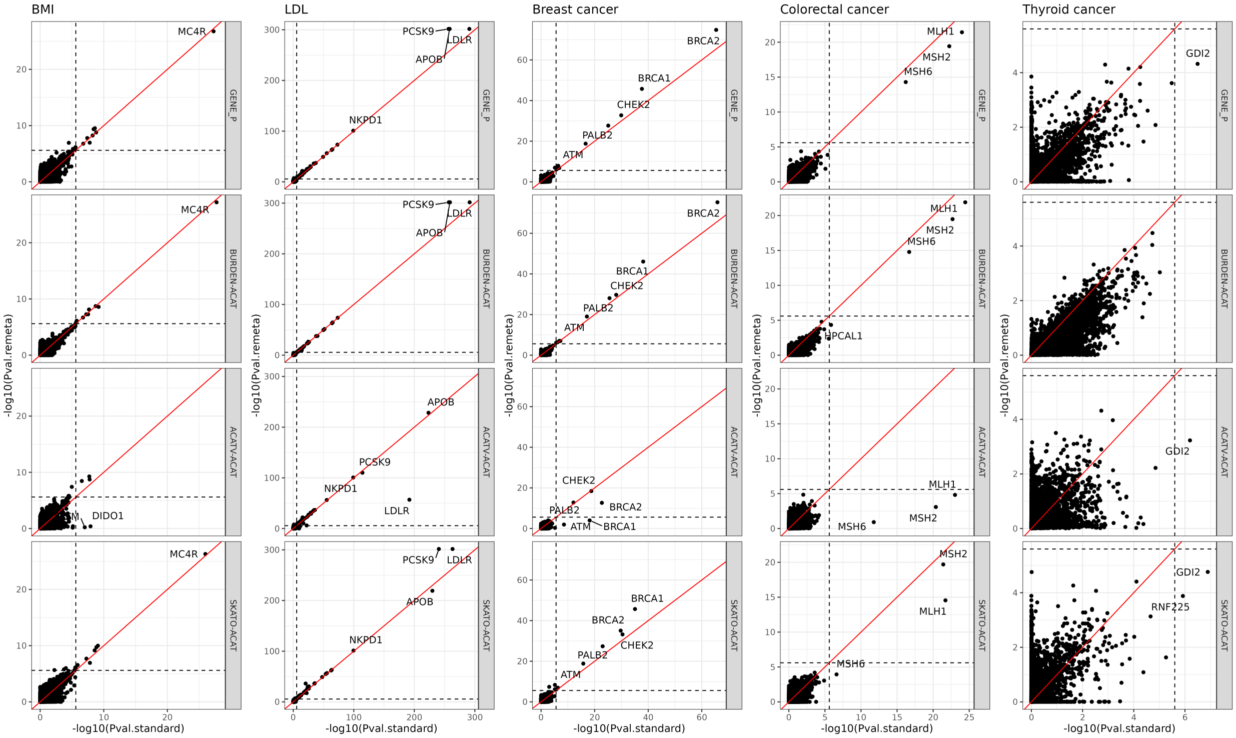

Supplementary Figure 15: Comparison of -log10 p-values between REMETA to standard meta-analysis across 5 traits. Meta-analysis was performed between 3 subsets of UK Biobank unequal sample sizes ($n_1 = 78,229$, $n_2 = 156,458$, $n_3 = 234,688$; a 1:2:3 ratio). Each point is a single gene, where multiple p-values per gene have been combined using ACAT. Four types of gene-based tests are compared: GENE P, burden, ACATV, and SKATO. *GENE P: Omnibus test combining p-values from BURDEN-ACAT, ACATV-ACAT, and SKATO-ACAT using ACAT. *BURDEN-ACAT: ACAT of burden meta-analysis p-values. *ACATV-ACAT: ACAT of ACATV meta-analysis p-values per gene. *SKATO-ACAT: ACAT of SKATO meta-analysis p-values per gene. Dashed lines correspond to a p-value of 2.5×10^{-6}.

3 List of investigators from the Regeneron Genetics Center

RGC Management & Leadership Team
Aris Baras, Gonçalo Abecasis, Adolfo Ferrando, Giovanni Coppola, Andrew Deubler, Luca A Lotta, John D Overton, Jeffrey G Reid, Alan Shuldiner, Katherine Siminovitch, Jason Portnoy, Marcus B Jones, Lyndon Mitnaul, Alison Fenney, Jonathan Marchini, Manuel Allen Revez Ferreira, Maya Ghoussaini, Mona Nafde, William Salerno, Cristen Willer, Lourdes Crane.

Sequencing & Lab Operations
John D Overton, Christina Beechert, Erin Fuller, Laura M Cremona, Eugene Kalyuskin, Hang Du, Caitlin Forsythe, Zhenhua Gu, Kristy Guevara, Michael Lattari, Alexander Lopez, Kia Manoochehri, Prathyusha Challa, Manasi Pradhan, Raymond Reynoso, Ricardo Schiavo, Maria Sotiropoulos Padilla, Chenggu Wang, Sarah E Wolf, Hang Du, Kristy Guevara.

Genome Informatics & Data Engineering
Jeffrey G Reid, Mona Nafde, Manan Goyal, George Mitra, Sanjay Sreeram, Rouel Lanche, Vrushali Mahajan, Sai Lakshmi Vasireddy, Gisu Eom, Krishna Pawan Punuru, Sujit Gokhale, Benjamin Sultan, Pooja Mule, Mudasar Sarwar, Muhammad Aqeel, Xiaodong Bai, Lance Zhang, Sean O'Keeffe, Razvan Panea, Evan Edelstein, Ayesha Rasool, William Salerno, Evan K Maxwell, Boris Boutkov, Alexander Gorovits, Ju Guan, Lukas Habegger, Alicia Hawes, Olga Krasheninina, Samantha Zarate, Adam J Mansfield, Lukas Habegger.

Analytical Genetics & Data Science
Gonççalo Abecasis, Manuel Allen Revez Ferreira, Joshua Backman, Kathy Burch, Adrian Campos, Liron Ganel, Sheila Gaynor, Benjamin Geraghty, Arkopravo Ghosh, Salvador Romero Martinez, Christopher Gillies, Lauren Gurski, Eric Jorgenson, Tyler Joseph, Michael Kessler, Jack Kosmicki, Adam Locke, Priyanka Nakka, Jonathan Marchini, Karl Landheer, Olivier Delaneau, Maya Ghoussaini, Anthony Marcketta, Joelle Mbatchou, Arden Moscati, Anita Pandit, Jonathan Ross, Carlo Sidore, Eli Stahl, Timothy Thornton, Sailaja Vedantam, Rujin Wang, Kuan-Han Wu, Bin Ye, Blair Zhang, Andrey Ziyatdinov, Yuxin Zou, Jingning Zhang, Kyoko Watanabe, Mira Tang, Frank Wendt, Suganthi Balasubramanian, Suying Bao, Kathie Sun, Chuanyi Zhang, Sean Yu, Aaron Zhang, David Corrigan, Dhruv Shidhaye, Chen Wang, Keyrun Adhikari, Alexander Lachmann.

Therapeutic Area Genetics
Adolfo Ferrando, Giovanni Coppola, Luca A. Lotta, Alan Shuldiner, Katherine Siminovitch, Brian Hobbs, Jon Silver, William Palmer, Rita Guerreiro, Amit Joshi, Antoine Baldassari, Cristen Willer, Sarah Graham, Ernst Mayerhofer, Erola Pairo Castineira, Mary Haas, Niek Verweij, George Hindy, Jonas Bovijn, Tanima De, Luanluan Sun, Olukayode Sosina, Arthur Gilly, Peter Dornbos, Juan Rodriguez-Flores, Moeen Riaz, Manav Kapoor, Gannie Tzoneva, Momodou W Jallow, Anna Alkelai, Ariane Ayer, Veera Rajagopal, Sahar Gelfman, Vijay Kumar, Jacqueline Otto, Jose Bras, Silvia Alvarez, Jessie Brown, Hossein Khiabanian, Joana Revez, Kimberly Skead, Valentina Zavala, Jae Soon Sul, Lei Chen, Sam Choi, Amy Damask, Nan Lin, Charles Paulding, Sameer Malhotra, Joseph Herman.

Research Program Management & Strategic Initiatives
Marcus B Jones, Michelle G LeBlanc, Nadia Rana, Jennifer Rico-Varela, Jaimee Hernandez, Larizbeth Romero, Ashley Paynter.

Senior Partnerships & Business Operations
Randi Schwartz, Lourdes Crane, Alison Fenney, Jody Hankins, Anna Han, Samuel Hart, Ryan Smith.

Business Operations & Administrative Coordinators
Ann Perez-Beals, Gina Solari, Johannie Rivera-Picart, Michelle Pagan, Sunilbe Siceron.
