# Supplementary material for: Computationally efficient meta-analysis of gene-based tests using summary statistics in large-scale genetic studies
Source: Nat Genet. 2025 Nov 12;57(12):3193–200. doi: 10.1038/s41588-025-02390-0 (PMC12695653; doi:10.1038/s41588-025-02390-0)
Supplement: Supplementary file 2 — Reporting Summary [file 41588_2025_2390_MOESM2_ESM.pdf]

nature portfolio

Corresponding author(s): Jonathan Marchini

Last updated by author(s): Aug 15, 2025

Reporting Summary

Nature Portfolio wishes to improve the reproducibility of the work that we publish. This form provides structure for consistency and transparency in reporting. For further information on Nature Portfolio policies, see our Editorial Policies and the Editorial Policy Checklist.

Statistics

For all statistical analyses, confirm that the following items are present in the figure legend, table legend, main text, or Methods section.

n/a	Confirmed	
☐	☒	The exact sample size (*n*) for each experimental group/condition, given as a discrete number and unit of measurement
☒	☐	A statement on whether measurements were taken from distinct samples or whether the same sample was measured repeatedly
☐	☒	The statistical test(s) used AND whether they are one- or two-sided<br>*Only common tests should be described solely by name; describe more complex techniques in the Methods section.*
☐	☒	A description of all covariates tested
☐	☒	A description of any assumptions or corrections, such as tests of normality and adjustment for multiple comparisons
☐	☒	A full description of the statistical parameters including central tendency (e.g. means) or other basic estimates (e.g. regression coefficient) AND variation (e.g. standard deviation) or associated estimates of uncertainty (e.g. confidence intervals)
☐	☒	For null hypothesis testing, the test statistic (e.g. *F*, *t*, *r*) with confidence intervals, effect sizes, degrees of freedom and *P* value noted<br>*Give P values as exact values whenever suitable.*
☒	☐	For Bayesian analysis, information on the choice of priors and Markov chain Monte Carlo settings
☒	☐	For hierarchical and complex designs, identification of the appropriate level for tests and full reporting of outcomes
☒	☐	Estimates of effect sizes (e.g. Cohen's *d*, Pearson's *r*), indicating how they were calculated

Our web collection on statistics for biologists contains articles on many of the points above.

Software and code

Policy information about availability of computer code

Data collection	No software was used for data collection
Data analysis	REMETA (version 0.9) https://github.com/rgcgithub/remeta<br>REGENIE (version 4.0) https://rgcgithub.github.io/regenie/<br>metaSTAAR (version 0.9.6.3) https://github.com/xihaoli/MetaSTAAR

For manuscripts utilizing custom algorithms or software that are central to the research but not yet described in published literature, software must be made available to editors and reviewers. We strongly encourage code deposition in a community repository (e.g. GitHub). See the Nature Portfolio guidelines for submitting code & software for further information.

Data

Policy information about availability of data

All manuscripts must include a data availability statement. This statement should provide the following information, where applicable:

- Accession codes, unique identifiers, or web links for publicly available datasets
- A description of any restrictions on data availability
- For clinical datasets or third party data, please ensure that the statement adheres to our policy

The individual-level genotype and phenotype data are available through formal application to the UK Biobank http://www.ukbiobank.ac.uk

Research involving human participants, their data, or biological material

Policy information about studies with human participants or human data. See also policy information about sex, gender (identity/presentation), and sexual orientation and race, ethnicity and racism.

Reporting on sex and gender	Sex of the study participants was collected as part of the UK Biobank study as described in Bycroft et al. Nature 2018 https://www.nature.com/articles/s41586-018-0579-z<br><br>54.2% of participants are women<br><br>Sex was used as a covariate in regression models for association testing
Reporting on race, ethnicity, or other socially relevant groupings	Genetic ancestry and continental ancestry assignments of each participant were inferred from the genotyping array and exome sequencing data as described in Backman et al, Nature 2021 https://www.nature.com/articles/s41586-021-04103-z.<br><br>To control for confounding due to genetic ancestry, ancestry-informative principal components (PCs) derived from genotyping array and exome sequencing data were used as covariates in association tests.
Population characteristics	Details can be found in Bycroft et al. Nature 2018 https://www.nature.com/articles/s41586-018-0579-z.<br>Briefly, 94.7% of participants are of European ancestry, 54.2% are female, the age at assessment is 58. Each participant reports 8 inpatient ICD10 3D codes on average.
Recruitment	Please see Bycroft et al. Nature 2018 https://www.nature.com/articles/s41586-018-0579-z
Ethics oversight	Ethical approval for the UK Biobank was previously obtained from the North West Centre for Research Ethics Committee (11/NW/0382). The work described herein was approved by the UK Biobank under application no. 26041.

Note that full information on the approval of the study protocol must also be provided in the manuscript.

Field-specific reporting

Please select the one below that is the best fit for your research. If you are not sure, read the appropriate sections before making your selection.

☒ Life sciences ☐ Behavioural & social sciences ☐ Ecological, evolutionary & environmental sciences

For a reference copy of the document with all sections, see nature.com/documents/nr-reporting-summary-flat.pdf

Life sciences study design

All studies must disclose on these points even when the disclosure is negative.

Sample size	We used genetic data on 469,376 samples who were participants of the UK Biobank. This dataset is one of the largest genetic datasets available and provides a sufficient test dataset for the new method described in the paper. We ran analysis on several different subsets of the datasets to evaluate different aspects of the method.
Data exclusions	Certain samples and genetic variants were excluded as part of the standard quality control pipeline applicable to the UK Biobank study. Details can be found in Bycroft et al. Nature 2018 https://www.nature.com/articles/s41586-018-0579-z and Backman et al, Nature 2021 https://www.nature.com/articles/s41586-021-04103-z, both of which are cited in the paper.
Replication	The purpose of this study is to describe the performance of the new method compared to other methods. We make no claim on new discoveries from the analysis, so there is no requirement for replication
Randomization	The purpose of this study is to describe the performance of the new method compared to other methods. We use real phenotypes on the UKB participants, so there is no requirement for randomization of subjects.
Blinding	The purpose of this study is to describe the performance of the new method compared to other methods, so blinding is not relevant to this study.

Reporting for specific materials, systems and methods

We require information from authors about some types of materials, experimental systems and methods used in many studies. Here, indicate whether each material, system or method listed is relevant to your study. If you are not sure if a list item applies to your research, read the appropriate section before selecting a response.

Materials & experimental systems

n/a	Involved in the study
☒	☐ Antibodies
☒	☐ Eukaryotic cell lines
☒	☐ Palaeontology and archaeology
☒	☐ Animals and other organisms
☒	☐ Clinical data
☒	☐ Dual use research of concern
☒	☐ Plants

Methods

n/a	Involved in the study
☒	☐ ChIP-seq
☒	☐ Flow cytometry
☒	☐ MRI-based neuroimaging

Plants

Seed stocks

Report on the source of all seed stocks or other plant material used. If applicable, state the seed stock centre and catalogue number. If plant specimens were collected from the field, describe the collection location, date and sampling procedures.

Novel plant genotypes

Describe the methods by which all novel plant genotypes were produced. This includes those generated by transgenic approaches, gene editing, chemical/radiation-based mutagenesis and hybridization. For transgenic lines, describe the transformation method, the number of independent lines analyzed and the generation upon which experiments were performed. For gene-edited lines, describe the editor used, the endogenous sequence targeted for editing, the targeting guide RNA sequence (if applicable) and how the editor was applied.

Authentication

Describe any authentication procedures for each seed stock used or novel genotype generated. Describe any experiments used to assess the effect of a mutation and, where applicable, how potential secondary effects (e.g. second site T-DNA insertions, mosiacism, off-target gene editing) were examined.
